# Cullin 4b-RING ubiquitin ligase targets IRGM1 to regulate Wnt signaling and intestinal homeostasis

**DOI:** 10.1038/s41418-022-00954-9

**Published:** 2022-02-23

**Authors:** Yujia Fan, Xiaohan Huo, Beibei Guo, Xiaohui Zhang, Yang Yang, Jiabei Lian, Xinyuan Meng, Yiwen Shao, Yongxin Zou, Haiyang Guo, Haitao Wang, Gongping Sun, Hao Dou, Jinshen Wang, Changshun Shao, Yaoqin Gong, Huili Hu

**Affiliations:** 1 The Key Laboratory of Experimental Teratology, Ministry of Education, Department of Molecular Medicine and Genetics, School of Basic Medical Sciences, Cheeloo College of Medicine, Shandong University, 250012 Jinan, China; 2 The Research Center of Stem Cell and Regenerative Medicine, School of Basic Medical Sciences, Cheeloo College of Medicine, Shandong University, 250012 Jinan, China; 3 Department of Clinical Laboratory, the Second Hospital of Shandong University, 250033 Jinan, China; 4 Department of Pathology, the Second Hospital of Shandong University, 250033 Jinan, China; 5 The Key Laboratory of Experimental Teratology, Ministry of Education, Department of Histoembryology, School of Basic Medical Sciences, Cheeloo College of Medicine, Shandong University, 250012 Jinan, China; 6 Department of Gastrointestinal Surgery Shandong Provincial Hospital Affiliated to Shandong First Medical University, Jinan, Shandong 250021 China; 7 The State Key Laboratory of Radiation Medicine and Protection, Institutes for Translational Medicine, Soochow University, 215123 Suzhou, China

**Keywords:** Development, Ubiquitin ligases

## Abstract

Hierarchical organization of intestine relies on the self-renewal and tightly regulated differentiation of intestinal stem cells (ISCs). Although signals like Wnt are known to sustain the continued intestinal renewal by maintaining ISCs activity and lineage commitment, molecular mechanisms underlying ISCs ‘stemness’ and supportive niche have not been well understood. Here, we found that CUL4B-RING ubiquitin ligase (CRL4B) regulates intestinal homeostasis by targeting immunity-related GTPase family M member 1 (IRGM1) for proteasomal degradation. CUL4B was mainly expressed at ISCs zone. Deletion of *Cul4b* led to reduced self-renewal of ISCs and a decreased lineage differentiation towards secretory progenitors through downregulated Wnt signals. Besides, *Cul4b*-null mice exhibited impaired Paneth cells number and structure. Mechanistically, CRL4B complex were associated with WD40 proteins and targeted IRGM1 at K270 for ubiquitination and proteosomal degradation. Impaired intestinal function caused by CUL4B deletion was rescued by down-regulation of its substrate IRGM1. Our results identified CUL4B as a novel regulator of ISCs and revealed a new 26 S proteasome degradation mechanism in intestine self-renewal and lineage commitment.

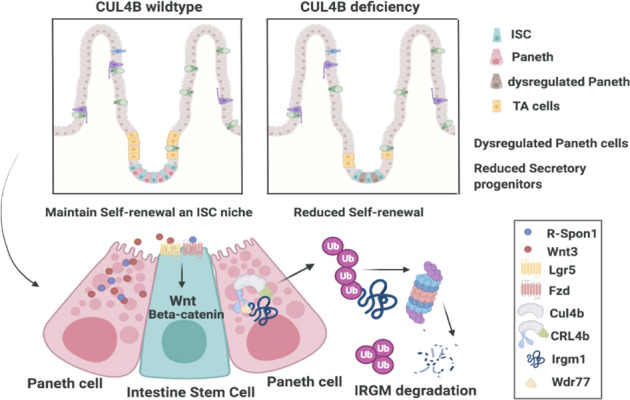

## Introduction

The mammalian intestine is organized into crypt-villi structures and self-renews with a rapid turnover rate of 4–5 days. Lgr5^+^ (also called crypt base columnar, CBC) cells at the bottom of crypt act as intestinal stem cells (ISCs) during intestinal homeostasis [1]. ISCs maintain continued self-renewing ability and produce differentiated progeny including rapidly proliferating transit-amplifying (TA) cells, post-mitotic enterocytes, absorptive and secretory lineages [2, 3]. Among them, larger, granule-containing Paneth cells intermingled with ISCs at the bottom crypt function as components of stem cell niche by secreting factors and host defensing [4, 5].

ISC stemness is tightly controlled by various pathways. Wnt signals restricted to the lower crypt form a gradient to fuel the continued renewal by maintaining ISC activity and regeneration [6, 7]. Lgr5, the marker of stem cells, is the direct target gene of Wnt signaling pathway [8]. Deletion of *Ctnnb1* and *Tcf7l2* (TCF4), the downstream transcription factors of Wnt pathway, in *pVillin*-Cre^ERT2^ mice results in a rapid loss of Lgr5^+^ cells [9]. Stem cell-specific activation of Wnt signals by *Apc* mutation confers a growth advantage [10, 11]. Conversely, overexpression of the secreted Wnt inhibitor Dickkopf1 (Dkk1) leads to the loss of crypts [12]. Paneth-cell derived Wnt3a and R-spondin1 are proved to be essential to maintain 3D organoids culture for ex vivo expansion [7]. Besides epithelial signals, reduced mTOR activity by calorie restriction or inhibition of glycolysis in Paneth cells metabolically also boosts ISC function [13]. The growth factors and cytokines secreted by mesenchymal cells also provide vital supporting niche for stem cells [14]. Thus identification of additional regulators of Wnt pathway in ISC niche will definitely contribute to a better understanding of ISC regulating network. Although loss of IRGM1 has been reported to result in disrupted intestinal epithelial function, gut dysbiosis, defects in anti-microbial peptide secretion and stress responses to pathogenic microbes [15, 16], however its role in ISC regulation has not been documented.

Cul4 encodes the scaffold protein of Cullin-Ring family, the biggest E3 ubiquitin ligase complex in mamallian cells [17]. CUL4B assembles DDB1, ROC1 and substrate receptors to form various CUL4B-RING E3 ligase complexes (CRL4B). CRL4B catalyzes polyubquitination of substrates for proteosomal degradation [18]. Although CUL4B possesses high homology with CUL4A, the nuclear localization sequences (NLS) of CUL4B distinguishes it from CUL4A in multiple biological processes [19]. We and other group have found that *CUL4B* mutation causes human X-linked intellectual disabilities [20, 21]. Disruption of *Cul4b* in mice results in early embryonic lethality before E9.5 [22, 23]. CUL4B in extraembryonic tissues is essential for early development. Selective deletion of CUL4B in the epiblast shows no significant defects except for the abnormal spermatogenesis [24]. Our recent study reveal the function of CUL4B in adipogenesis [25], but no research has addressed its role in intestine regulation.

We here studied the function of CUL4B in intestinal homeostasis. Our results showed a surprising cytoplasm-specific localization of CUL4B at the ISCs zone. To determine the function of CUL4B in the intestine, we generated conditional knockout mice and in vitro organoids to investigate how CUL4B modulates ISC stemness and cell lineage commitment. We found that CUL4B was required for intestine self-renewal and proliferation. Loss of *Cul4b* caused scarcity of Lgr5^+^cells, Paneth cells and secretory lineages by decreasing Wnt signals. We uncovered IRGM1 as the ubiquitination substrate of CRL4B. CRL4B ubiquitylated IRGM1 at K270 and promoted Wnt3 secretion. Our findings suggest that CUL4B is essential in maintaining ISCs stemness and intestinal homeostasis.

## Materials and methods

### Animals and administration

All animal experiments were approved by the Animal Care and Use Committee of the School of Basic Medical Science of Shandong University (No. LL-201502043 and ECSBMSSDU2019-2-008). *Lgr5*-EGFP-ires-Cre^ERT2^, *CAG*-Cre^ERT2,^ and Rosa26-LSL-Cas9-tdTomato mice were from the Jackson Laboratory (stock number 008875 and 004682) or GemPharmatech co. Ltd (stock numbers T002249). *pVillin*-Cre mice were provided by Dr. Baichun Jiang. *Cul4b* transgenic mice were generated as described previously [26]. Mice were housed in SPF facilities. The wildtype or *Cul4b* knockout mice from 8 to16 weeks were used for most experiments. For Cre induction, mice with the genotyping of *Lgr5-*EGFP-ires-Cre^ERT2^ and *CAG-*Cre^ERT2^ were intraperitoneally injected with 100 μl tamoxifen at the concentration of 2 mg/ml for 5 consecutive days.

### Tissue histology and immunofluorescence

Small intestines or colons from WT or KO mice were isolated, rinsed with cold PBS, fixed in 4% paraformaldehyde overnight at 4 °C, and embedded in Tissue-Tek® OCT or paraffin. Tissues were cut into 4 μm sections. Unstained paraffin sections were washed twice with xylene dewax, dehydrated in ethanol solution with decreasing concentration (100%, 95%, 90%, 80%, 75%, 70%, 50%, 25%), and stained with hematoxylin-eosin staining (H&E). To assess tissue histology, the crypts and villus length were quantified from crypt/villus units per mouse with triplicates. Positive staining cells with PCNA, Olfm4, or Lyz were quantified from approximately 50 well-oriented crypt/villus units per mouse with triplicates.

Unfixed tissue segments were embedded with Tissue-Tek® OCT and frozen in freezing table. Sections were cut on a Freezing Microtomes. For immunofluorescence, antigen retrieval was achieved by boiling in Citrate Antigen Retrieval Solution. Sections were washed in PBS, then permeabilized (0.2% Triton X-100 in PBS) and incubated in blocking solution (10% v/v goat/donkey serum in PBS) at 37 °C for one hour. Primary antibody treatment was performed overnight at 4 °C in blocking solution. Secondary antibody was incubated one hour at room temperature. Microscopic images were obtained with Laser Confocal Microscope (LSM880, LSM900) and OLYMPUS imaging.

The primary antibodies included the following: anti-CUL4B (Sigma, St. Louis, MO, USA, 1:750), anti-Lyz (Abcam, Cambridge, UK, 1:1000), anti-Ki67 (Abcam, 1:500), anti-PCNA (GeneTex, Alton PkwyIrvine, CA, USA, 1:500), anti-β-catenin (Santa Cruz, CA, USA, 1:1000), anti-GFP (Rockland, USA, 1:500), anti-Olfm4 (Cell signaling, Beverly, MA, USA 1:400), anti-BrdU (Abcam, 1:1000), anti-UEA-1 (Sigma, 1:200), anti-Mucin2 (Santa, 1:100), anti-ChgA (Santa, 1:100), anti-Villin(Abcam, 1:400) and anti-WDR77(Abcam, 1:200). Secondary antibodies included peroxidase-conjugated goat anti-rabbit IgG, goat anti-mouse IgG (Jackson ImmunoResearch, West Grove, PA, USA, 1:200) and donkey anti-goat IgG (Invitrogen, Carlsbad, CA, USA, 1:500).

### Western blot

Proteins were extracted from mouse tissue and organoids. 25 micrograms of protein were immunoblotted by standard protocols. The primary antibodies included as the following: anti-CUL4B (Sigma), anti-Lgr5 (BBI Life Sciences, Shanghai, China), anti-Histone (GeneTex), anti-α-Tubulin (Proteintech, Wuhan, China), anti-Cytokeratin 20 (GeneTex), anti-GAPDH, anti-pGSK3β(Ser9), anti-p-β-catenin (S33/37/T41), anti-Non-p-β-catenin, anti-Ub (all antibodies from Cell signaling), anti-IRGM1(GeneTex, Cell signaling), anti-PTGES3 (Proteintech), anti-SLC5A1, anti-ABCG2, anti-STX18 (ORIGENE, Rockville, USA), anti-EPCAM (Abways, Shanghai, China), anti-ACTIN, anti-DDB1, anti-β-catenin (Santa Cruz), anti-HA (Rockland) anti-ROC1 and anti-WDR77, anti-WDR1(Abcam). The secondary antibodies included anti-rabbit and anti-mouse horseradish peroxidase (HRP) (Jackson ImmunoResearch, West Grove, PA, USA; 1:10000; 1:5000). The detection reagent Cheminoluminiscent Substrate was provided from the ECL kit (Thermo, USA). The band signals from Western blot results were analyzed by Volume Analysis of Quantity One with volume background subtraction (Bio-Rad, USA).

### Lineage tracing of Crypt–Villus units

For BrdU label assay, *Lgr5*-EGFP-ires-Cre^ERT2^; *Cul4b*^-/Y^ and *Lgr5*-EGFP-ires-Cre^ERT2^; *Cul4b*^fn/Y^(KO^Lgr5^) mice were abdominally injected with tamoxifen for 5 consecutive days. Each mouse was injected with 1 mg BrdU. On the 3^rd^ day after injection, small intestines were collected and analyzed by immunofluorescence. For self-renewal analysis, mice were injected with 0.1 mg tamoxifen per gram. SI were collected and analyzed from *Lgr5*-EGFP-ires-Cre^ERT2^; *Cul4b*^-/Y^; Rosa26-LSL-Cas9-tdTomato; KI/KI and *Lgr5*-EGFP-ires-Cre^ERT2^; *Cul4b*^fn/Y^; Rosa26-LSL-Cas9-tdTomato; KI/KI (KO^Lgr5-Tom^) mice on the 3^rd^ day after tamoxifen injection.

### Organoids isolation, culture and passage

The crypt isolation and organoid culture were performed as described previously [11]. Crypts were released from small intestine by incubating with 2 mM EDTA in DPBS (on ice for 30 mins). Crypts were re-suspended with cold Advanced DMEM/F12 medium. Crypts were mixed with Matrigel (Corning, USA), and plated in 24-well plates. The basal differentiation culture medium (Differentiation Medium, DM) included: Advanced DMEM/F12 supplemented with 1×penicillin/streptomycin, 1×HEPES(Invitrogen), 1×Glutamax (Invitrogen), 1×B27 (Invitrogen), and 1 mmol/L N-acetylcysteine (Sigma-Aldrich) with 50 ng/mL recombinant epidermal growth factor (PepRotech, USA), Noggin (conditioned medium, 5% v/v), and R-spondin 1 (conditioned medium, 10% v/v). Expansion medium (EM) included DM plus 15% Wnt3a conditioned medium. The Cre expression was induced with treatment of 10 μM 4-OHT (Sigma-Aldrich) in organoids.

### Whole mount staining

Organoids were thoroughly removed from Matrigel with Cell Recovery Solution (Corning) on ice before centrifugation at the speed of 800 rpm. 4% paraformaldehyde was used to fix the organoids overnight at 4 °C. Immunofluorescence proceeded as outlined above. Microscopic observation was performed using a high-speed confocal platform (Andor, Dragonfly 200).

### Constructions and transfections

The ORF of mouse *Irgm1* (Myc-DDK-tagged) and *Cul4b* (Myc-DDK-tagged) were purchased from Origene and cloned into the lentiviral expression vector (pCDH-CMV-MCS-EF1-Puro, one kind gift of Dr. Jupeng Yuan, Shandong Cancer Hospital and Institute, China) or the His-Tag lentiviral expression vector (pLent-EF1a-FH-CMV-GFP-P2A-puro purchased from vigene).

For transfection, HEK293T cells were cultured to the concentration of 30%. Transfection was performed in 500 μl opti-DMEM with 20 μl siRNA or control with addition of 30 μl X-tremeGene (Roche, Mannheim, Germany) according to the manufacturer’s instruction. 2 μg constructions for ectopic expression of CUL4B, IRGM1 and HA-Ubiquitin were transfected by PEI transfection reagent (Merck) as indicated respectively. After 48 h incubation, MG132 was added at a final concentration of 10 μM. The cells were then harvested after 3 h incubation.

### RNA isolation and qRT-PCR analysis

The RNA was isolated with Trizol reagents (Invitrogen) or by isolation Kit (Qiagen, Dusseldorf, Germany) according to the manufacturer’s instruction. RNA was reversely transcribed into cDNA by reverse transcriptase (Thermo Scientific). Real Time PCR analysis was performed using gene-specific primers as listed in Table S1 and SYBR green master mix (Roche).

### RNA-seq and data analysis

RNA was prepared for transcriptome sequencing. Library was prepared and sequenced by Novogene. The sequencing data could be reached in GSE157818. Sequencing results were mapped and differential expression analysis of comparing groups (2–4 biological replicates per group) was performed using the DESeq2 R package (1.16.1). Corrected P-value of 0.05 and absolute fold change of two were set as the threshold for significantly differential expression.

Gene Set Enrichment Analysis (GSEA) analysis was performed by the GSEA analysis tool http://www.broadinstitute.org/gsea/index.jsp. GO, KEGG, Reactome, DO, DisGeNET data sets were used independently. We ranked GSEA database genes by their association with differential genes in crypts from WT (*N* = 4) and KO^IEC^ (*N* = 4) groups, organoids from WT (*N* = 2) and KO^IEC^ (*N* = 2) and organoids from WT (*N* = 2) and KO^CAG^ (*N* = 2) groups.

### Differentiated protein/ubiquitylated protein identification and quantification by LC-MS/MS

Crypts from WT (*N* = 3) and KO^IEC^ (*N* = 3) were isolated and mixed together. Differentiated proteins and differentiated ubiquitylated proteins were identified by Liquid Chromatography-Mass Spectrometry (LC-MS) in PTM Biolab LLC and analyzed by PTM bioinformatics Team. MS data in PRIDE with the number of PXD-021528. Venn diagram was performed to analyze the overlapping differentiated proteins that both downregulated in ubiquitylation and accumulated at protein level.

### Protein stability assay and analysis

HEK293T cells were transfected with Myc-Irgm1 (2 μg). 24 h after transfection, cells were treated with 240 μg/ml cycloheximide (CHX) to inhibit protein synthesis. CHX-treated cells were harvested at different time points (0, 16, 32, 48 h) and processed for immunoblotting with anti-Irgm1 antibody. Anti-ACTIN antibody was used as the internal control. Signals from the Western blots were analyzed by Volume Analysis of Quantity One (Bio-Rad) with background subtraction.

### FISH

The small intestine tissue was collected, cleaned, and then immediately put in the fixed fluid (DEPC) for 12 h. The tissue was dehydrated by gradient alcohol with paraffin embedding. The slices were boiled in the retrieval solution for 10–15 mins and naturally cooled. The objective tissue was marked, treated with proteinase K (20 μg/ml) solution and incubated at 37 °C. After washing, the section was incubated in a humidity chamber and hybridized with Lgr5 probe diluted in hybridization solution overnight. After hybridization, the sections were washed in 2×SSC for 10 mins at 37 °C.

### Protein purification and pull-down assays

pGEX4T3-WDR77 and pRsfduet-IRGM1 were individually expressed in *E.coli* and purified using glutathione-sepharose (GE Healthcare) or Ni^2+^-NTA (GE Healthcare). The purified GST-WDR77 and His-IRGM1 were stored in protein buffer containing 25 mM Tris7.6, 150 mM NaCl, and 1 mM DTT. The pull-down assays were performed by mixing GST-WDR77 and His-IRGM1 proteins. The mixture was then incubated with anti-WDR77 antibody (Abcam) and protein A/G sepharose (Santa Cruz) for 2 h at 4 °C. Immunoprecipitates were boiled in sample loading buffer for 5 mins and detected by Western blot.

### Ubiquitination assay

HEK293T cells were first transfected with indicated plasmids or siRNAs and treated with MG132 (20 μM). Cells were then harvested with PBS containing 10 mM NEM to prevent deubiquitination. Cells were lysed in cell lysis buffer containing 10 μM Tris 8.0, 150 mM NaCl, and 2% SDS by boiling the samples for 10 mins, followed by sonication (8 s, 1 cycle). Lysed sample supernatants were then incubated with anti-IRGM1 antibody (Cell Signaling Technology), mixed with protein A/G sepharose (Santa Cruz) for 12 h at 4 °C, followed by Western blot.

### In vitro ubiquitination assay

His-IRGM1 was expressed in *E.coli* and purified with Ni^2+^-NTA (GE Healthcare). His-IRGM1 was incubated with 200 ng E1 (UBE1), 500 ng E2 (UbcH5c), 10 μg His-Ub and 2 mM ATP (Enzo life sciences). The reaction was performed in the absence and presence of Flag-CUL4B co-IP products and incubated at 37 °C for 1 h. Samples were quenched in 6 M guanidinium-HCl (pH=8) containing 5 mM NEM. His-ubiquitinated proteins were pulled down with Ni^2+^-NTA (GE Healthcare), washed and eluted in sample buffers as described previously [27]. The mixture was then boiled in loading dye at 95 °C for 10 mins to disrupt the protein-protein interactions, followed by Western blot.

### Immunoprecipitation

Protein supernatants were incubated with indicated antibodies and protein A/G sepharose (Santa Cruz) for 2 h at 4 °C. Immunoprecipitates were boiled in sample loading dye at 95 °C for 5 mins, followed by Western blot.

### Wnt luciferase activity and conditional medium assay

For Wnt luciferase activity, medium was collected from wildtype or knockout organoids. HEK293 STF cells were treated with the medium for 48 h and luciferase was detected. For conditional medium assay, wildtype organoids were digested into single cells and plated at the concentration of 1 × 10^4^ cells per well in 96-well plates. After 24 h, the culture medium was changed with conditional medium collected from either the wildtype or the knockout organoids. The conditional medium was produced by the same number of wildtype or the knockout organoids after for 24 h and 48 h culturing. RNA was collected or cellular ATP activity and RNA levels were measured.

### Statistical analysis

Statistical analyses were conducted with Prism software (GraphPad, La Jolla, CA). For pairwise and two independent group comparison two-tailed t-test was used. Data are presented as mean ± SEM and *P* values determined by Student t-test; *P < 0.05. **P < 0.01. ***P < 0.001. ****P < 0.0001 was considered statistically significant.

## Results

### CUL4B is highly expressed in mouse intestinal crypts

**Fig. 1:**
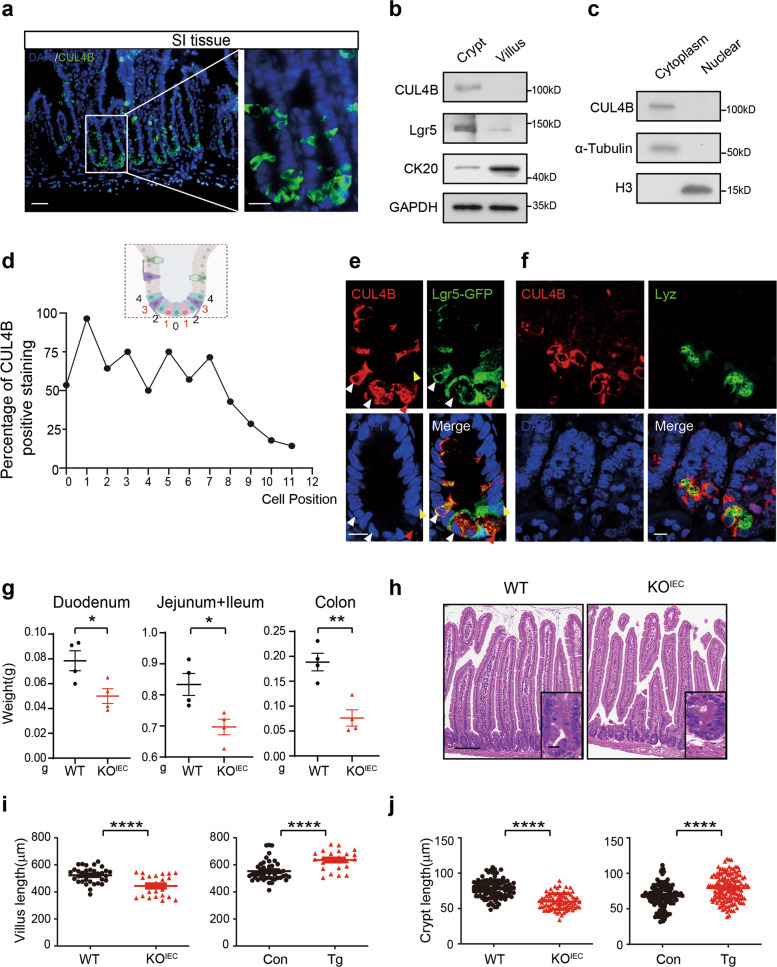
Loss of CUL4B at the crypt base disturbs intestinal cryp-villi structure. **a** The representative images of CUL4B staining on small intestine (SI) tissue. Green, CUL4B, Blue, DAPI. The scale bar is 50 μm (Left) and 20 μm (Right). **b** Western blots analysis of CUL4B, the stem cell marker (Lgr5), and the villi marker (CK20) in extracts from crypt and villus of the SI. **c** Western blots analysis of CUL4B, the nuclear marker (Histone protein, H3), and the cytoplasm marker (α-Tubulin) in cytoplasmic and nuclear extracts from crypts of the SI. **d** The percentage of CUL4B positive staining by statistical quantification of cell position along the crypt-villus from the bottom. **e** The representative image of co-staining of Lgr5 and CUL4B in the crypts of *Lgr5*-EGFP-ires-Cre^ERT2^ mouse. The white arrowhead, co-expression of Lgr5 and CUL4B. The red arrowhead, exclusive expression of CUL4B. The green arrowhead, exclusive expression of Lgr5. The scale bar is 10 μm. **f** The representative image of co-staining of Lyz and CUL4B in the crypts. The scale bar is 50 μm. **g** The measurement of intestine weight of duodenum, jejunum plus ileum, colon of KO^IEC^ (*N* = 4), and WT mice (*N* = 4). Error bars represent standard errors. **P < 0.01, *P < 0.05. **h** The representative images of H&E staining of sections of SI from WT and KO^IEC^ mice. The scale bar is 100 μm(left), 25 μm(right). **i**, **j** The Measurement of villus length (**i**) and crypt length (**j**) of KO^IEC^, Tg and WT mice (*N* = 3). Error bars represent standard errors. ****P < 0.0001.

To investigate the role of CUL4B in intestinal regulation, we firstly determined the expression pattern of CUL4B in intestine. As shown in Fig. 1a, CUL4B was highly expressed at the bottom of crypts where located the stem cell compartment. Interestingly, unlike many cell types in other mammalian tissues where CUL4B was predominantly localized in the nucleus, CUL4B was mainly detected in the cytoplasm of intestine (Supplementary Fig. S1a and Movie S1). Western blot analysis confirmed its cytoplasmic location at crypts as well (Fig. 1b, c). The statistical analysis of immunofluorescent positive staining at each cell position showed that CUL4B expression was gradually decreasing along the crypt-villi axis (Fig. 1d). In accordance with it, co-staining showed the co-express of CUL4B with Lgr5^+^ ISCs and Lyz^+^ Paneth cells (Fig. 1e, f). These results above indicate that CUL4B is highly expressed in bottom crypt, suggesting a potential role of CUL4B in intestine regulation.

### Loss of CUL4B in intestinal epithelial cells retards intestinal development

To investigate the function of CUL4B in intestine, we generated the mice with intestinal epithelium specific deletion of *Cul4b* (*pVillin*-Cre; *Cul4b*^fn/Y^, abbreviated as KO^IEC^) and compared them with the wildtype control (*Cul4b*^fn/Y^, abbreviated as WT). Knockout efficiency of KO^IEC^ mice was confirmed at the mRNA and protein levels (Supplementary Fig. S1b, c). KO^IEC^ mice were born at the expected Mendelian ratios. Epithelial deficiency of CUL4B had no effect on mouse body weight but led to decreased intestinal weight, shortened villi, and a reduction of crypt cell number (Fig. 1g–j and Supplementary Fig. S1d). To confirm the role of CUL4B in regulating intestinal development, a previously generated CUL4B overexpression model of transgenic mice were further evaluated (EGFP-CUL4B under the promoter of CMV, abbreviated as Tg) [26]. As expected, we observed enlongated crypts and villi in the transgenic mice (Fig. 1i, j and Supplementary Fig. S1e). Our results suggest that CUL4B promotes crypt-villi formation and intestinal development.

### CUL4B is essential to maintain intestinal self-renewal in vivo and vitro

**Fig. 2:**
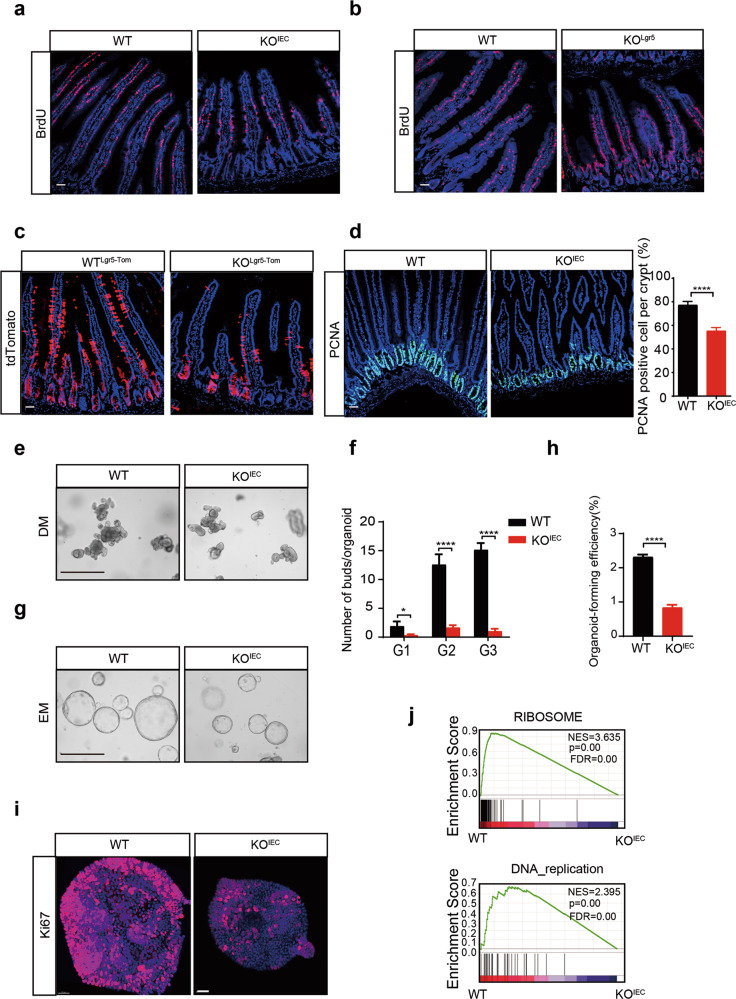
*Cul4b* is essential for homeostatic self-renewal and proliferation. **a** BrdU label assay shows decreased self-renewal rate in KO^IEC^ mice. The scale bar is 50 μm. **b** BrdU label assay shows decreased self-renewal rate in KO^Lgr5^ mice 3 days after tamoxifen induction administration. The scale bar is 50 μm. **c** Lineage tracing of Lgr5^+^ positive cells and their ascents (tdTomato) in the jejunum of KO^Lgr5-Tom^ mice and control 3 days after tamoxifen induction. The scale bar is 50 μm. **d** The representative images and quantification of PCNA positive staining cells of WT and KO^IEC^ mice (*N* = 3). The scale bar is 50 μm. ****P < 0.0001. **e** The representative images of organoids of passage 2 derived from WT and KO^IEC^ mice cultured in differentiation medium (DM). The scale bar is 400 μm. **f** The average number of buddings per organoid derived from WT and KO^IEC^ mice (*N* = 3). Error bars represent standard errors. *P < 0.05, ****P < 0.0001. **g** The representative images of organoids at passage 2 derived from WT and KO^IEC^ mice cultured in expansion medium (EM). The scale bar is 400 μm. **h** Colony-forming effciency of organoids cultured in expansion medium from KO^IEC^ and WT mice. Error bars represent standard errors. ****P < 0.0001. **i** Whole-mount staining of Ki67 in intestinal organoids from WT and KO^IEC^ mice. The scale bar is 50 μm. **j** GSEA analysis for differentially expressed genes between KO^IEC^ and WT crypts. Ribosome and DNA replication pathways were enrichment in WT group. NES, normalized enrichment score; FDR, false discovery rate.

To determine the mechanisms underlying shortened intestine led by CUL4B defiency, we firstly examined intestinal renewal. 5-bromo-2-deoxyuridine (BrdU) labeling assay, which marks cycling crypt cells and their progenies, was performed. High resolution images revealed a lower self-renewal speed in both KO^IEC^ and KO^Lgr5^ mice (*Lgr5*-EGFP-IRES-Cre^ERT2^; *Cul4b*^fn/Y^, deteltion of CUL4B in Lgr5^+^ stem cells, abbreviated as KO^Lgr5^) compared with the WT control (Fig. 2a, b). We then crossed the KO^Lgr5^ with Rosa26-tdTomato mice to generate *Lgr5*-EGFP-IRES-Cre^ERT2^; *Cul4b*^fn/Y^; Rosa26-tdTomato mice (abbreviated as KO^Lgr5-Tom^). This strategy allowed for the tracking of Lgr5^+^cells by tdTomato by one pulse tamoxifen treatment. Three days after tamoxifen administration, the tdTomato-positive descendants extended markedly along the crypt-villus axis to the top in the wildtype, while most tdTomato-positive cells of KO^Lgr5-Tom^ mice still localized in the crypt compartment (Fig. 2c). PCNA staining showed a slower proliferation while cell apoptosis was not affected in KO^IEC^ mice (Fig. 2d and Supplementary Fig. S2a). Our findings revealed that CUL4B is indispensable to maintain intestinal renewal in vivo.

We next generated organoids to evaluate the self-rewal capacity of ISCs ex vivo. CUL4B expression was obviously elevated in organoids cultured in stem cell-enriched expansion medium (EM) than those in differetiation medium (DM) (Supplementary Fig. S2b). Organoids derived from KO^IEC^ mice displayed reduced budding efficiency during passaging (Fig. 2e, f). Decreased size and formation efficiency were also observed in KO^IEC^ organoids cultured in EM in single cell assay (Fig. 2g, h and Supplementary Fig. S2c). Meanwhile immunofluorescent staining of proliferative signal, Ki67 was obviously reduced (Fig. 2i and Supplementary Movie S2 and S3). To investigate the molecular alteration caused by loss of CUL4B, we then performed mRNA sequencing (RNA-seq) of isolated crypts (4KO^IEC^ vs 4WT) and in vitro cultured organoids (2KO^IEC^ vs 2WT). A tamoxifen-induced CUL4B knockout organoid model was also established from CAG-Cre^ERT2^; *Cul4b*^fn/Y^ mice and used for sequencing (2KO^CAG^ vs 2WT) after tamoxifen treatment. Differentially expressed genes (DEGs) in CUL4B-deficient crypts and organoids were identified in comparison with the wildtype respectively (All DEGs were listed in Supplementary Table S2). The gene set enrichment analysis (GSEA) analysis showed that DNA replication and ribosome-associated genes were significantly downregulated in both *Cul4b* knockout tissues and organoids (Fig. 2j and Supplementary Fig. S2d). Taken together, our data suggest that CUL4B is required for the maintenance of intestinal self-renewal.

### CUL4B deficency leads to dysregulated ISC differentiation

**Fig. 3:**
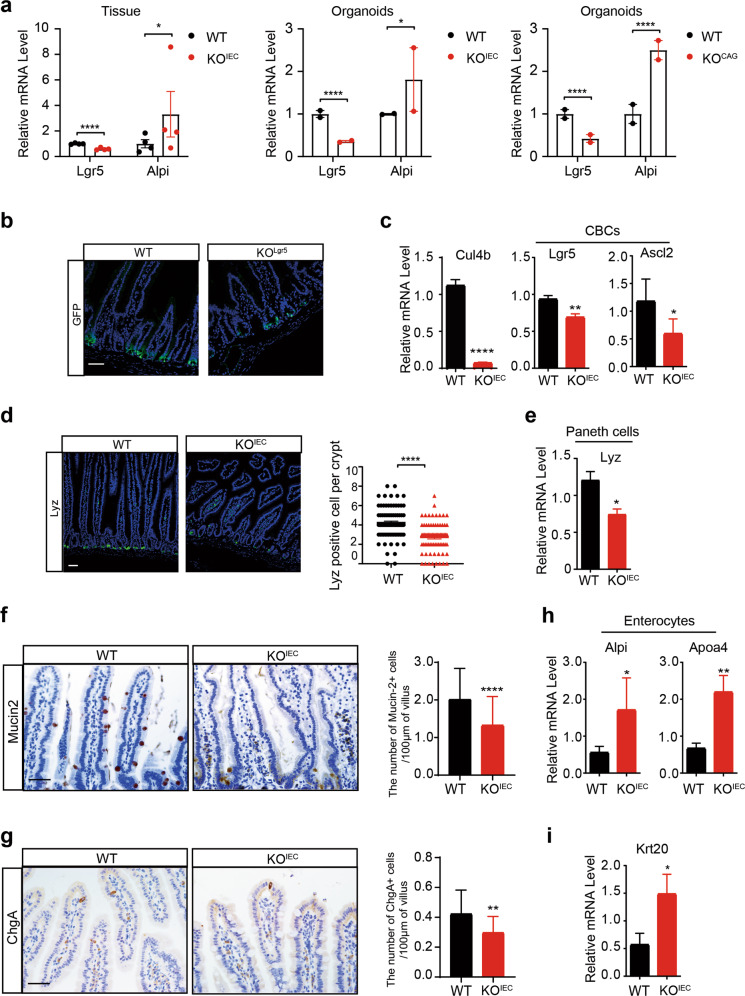
Lgr5^+^ stem cells and secretory lineage differentiation are reduced after CUL4B ablation. **a** The mRNA expression level of *Lgr5* and *Alpi* isolated from KO^IEC^ intestine crypts relative to WT littermates, KO^IEC^ organoids relative to WT and KO^CAG^ organoids relative to WT. *P < 0.05, ****P < 0.0001. **b** The representative images of GFP staining in SI of KO^Lgr5^ mice and WT littermates. (*N* = 3). The scale bar is 50 μm. ****P < 0.0001. **c** The mRNA expression level of stem cell markers in KO^IEC^ crypts relative to WT littermates. *P < 0.05, **P < 0.01, ****P < 0.0001. **d** The representative images of Lyz staining in SI of KO^IEC^ mice and WT littermates (*N* = 3). The scale bar is 50 μm. ****P < 0.0001. **e** The mRNA expression level of Lyz in KO^IEC^ crypts relative to WT littermates. *P < 0.05. **f** The representative images and quantification of Mucin2 staining in SI of KO^IEC^ and WT mice (*N* = 3). The scale bar is 50 μm. ****P < 0.0001. **g** The representative images and quantification of ChgA staining in SI of KO^IEC^ mice and WT littermates (*N* = 3). The scale bar is 50 μm. **P < 0.01. **h** The mRNA level of enterocytes markers *Alpi* and *Apoa4* in KO^IEC^ crypts relative to WT littermates. *P < 0.05, **P < 0.01. **i** The mRNA level of *Krt20* in KO^IEC^ crypts relative to WT littermates. *P < 0.05. Error bars represent standard errors in the figure.

We then checked RNA-seq data and performed immunostaining of intestinal cell lineage markers to identify the change of SI cell components after loss of CUL4B. Generally, RNA-seq data suggest Lgr5 was significantly down-regulated and differentiated enterocyte marker *Alpi* was increased in all three kinds of CUL4B deletion models (Fig. 3a). A decreased presence of ISCs was observed by GFP staining in KO^Lgr5^, Lgr5-FISH and Olfm4 staining in KO^IEC^ intestines (Fig. 3b and Supplementary Fig. S3a, b). Consistently, the ISCs markers such as Lgr5 and Ascl2 were down-regulated in KO^IEC^ crypts (Fig. 3c). For differentiated cell types, immunofluorescent and immunochemical staining showed CUL4B-deficient resulted in the reduction of Paneth cells (Lyz staining, Fig. 3d), Goblet cells (Mucin2 and Alcian Blue staining, Fig. 3f and Supplementary Fig. S3c) and enteroendocrine cells (ChgA^+^, Fig. 3g) in intestines, and the increased staining of Alkaline phosphatase (Supplementary Fig. S3d). qRT-PCR assay confirmed that Paneth cell marker *Lyz* was down-regulated (Fig. 3e), whereas the differentiated enterocyte markers such as *Alpi*, *Apoa4*, and *Krt20* were increased in *Cul4b*-deficient intestine (Fig. 3h, i). Mucin2^+^ staining and stem cell markers like *Lgr5* and *Ascl2* were also decreased in the colon of KO^IEC^ mice (Supplementary Fig. S3e, f). GO analysis revealed that the functions associated with absorption and digestion responsible by enterocytes was strengthened in KO^IEC^ intestine (Supplementary Fig. S3g, h). The peroxisome proliferator-activated receptor (PPAR) signal was also enriched in KO^IEC^ mice by GSEA analysis (Supplementary Fig. S3i). Taken together, these results indicate that CUL4B deficiency in intestinal epithelial cells compromise lineage development. Intestinal CUL4B promotes cell commitment toward secretory progenitors and their terminally differentiated lineages including Paneth cells, Goblet cells and enteroendocrine cells. Meanwhile, terminally differentiated lineage of enterocytes from absorptive progenitors was inhibited (Supplementary Fig. S3j).

### CUL4B regulates intestine through Wnt signals

**Fig. 4:**
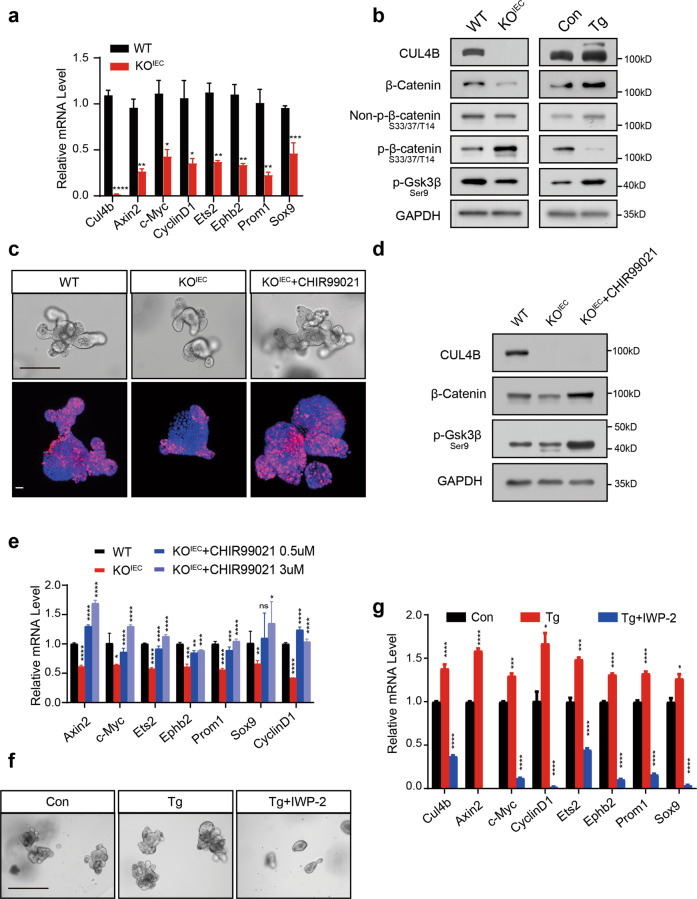
Impaired self-renewal after CUL4B ablation is due to disturbed Wnt signals. **a** The mRNA levels of Wnt/β-catenin target genes in KO^IEC^ crypts compared with WT control. *P < 0.05, **P < 0.01, ***P < 0.001, ****P < 0.0001. **b** The protein level of Wnt/β-catenin pathway was determined by Western blot in SIs organoids from KO^IEC^ and WT mice (Left), Tg and Con (Right). **c** The representative images in bright field (Upper) and Ki67 staining (Lower) of KO^IEC^ intestinal organoids with or without 3 μM CHIR99021 treatment compared with the WT. The scale bar is 100 μm (Upper) and 15 μm (Lower). **d** The protein level of Wnt/β-catenin pathway was determined by Western blot in SIs organoids from KO^IEC^ mice with or without 3μM CHIR99021 treatment. **e** The mRNA levels of Wnt/β-catenin target genes in KO^IEC^ and WT intestinal organoids with or without treatment with CHIR99021 (0.5 μM or 3 μM). *P < 0.05, **P < 0.01,***P < 0.001, ****P < 0.0001, ns, no significance. **f** The representative images in bright field of Tg intestinal organoids with or without treatment of 3 μM IWP-2 compared with Con. The scale bar is 400 µm. **g** The mRNA levels of Wnt/β-catenin target genes in Tg intestinal organoids with or without 3 μM IWP-2 treatment compared with Con. *P < 0.05, ***P < 0.001, ****P < 0.0001. Error bars represent standard errors in the figure.

Wnt signaling is essential for ISC self-renewal, proliferation of progenitors and transit-amplifying (TA) cells, as well as commitment toward secretory progenitors [28]. The fact that loss of CUL4B impaired intestinal self-renewal and secretory lineage differentiation prompted us to check whether CUL4B regulated intestine through Wnt signaling pathway. We examined the effect of CUL4B deficiency on the expression of the Wnt/beta-catenin target genes. qRT-PCR confirmed that lack of CUL4B resulted in a significant decrease of Wnt target genes (Fig. 4a). Western blot showed that decreased β-catenin, p-GSK3β (Ser9) and activating form of p-β-catenin (S33/37/T41) in *Cul4b* knockout mice. Consistently, they were increased in *CUL4B* transgenic intestine (Fig. 4b). These data indicate that decreased Wnt signaling could be responsible for the impaired intestinal renewal led by *Cul4b* deletion. Indeed, rescue attempt with exogenous administration of GSK3β inhibitor, CHIR99021, could efficiently block decreased budding formation and proliferation (Fig. 4c), the reduction of β-catenin (Fig. 4d) and its target genes (Fig. 4e) as well. Furthermore, treating CUL4B overexpression organoids with Wnt inhibitor IWP-2 significantly attenuated increased budding formation and expression of Wnt target genes (Fig. 4f, g). Altogether, these results suggest that decreased Wnt signaling is responsible for the impaired intestinal regulation caused by *Cul4b* deletion.

### Lack of CUL4B in intestinal epithelial cells impairs their secretory phenotype

**Fig. 5:**
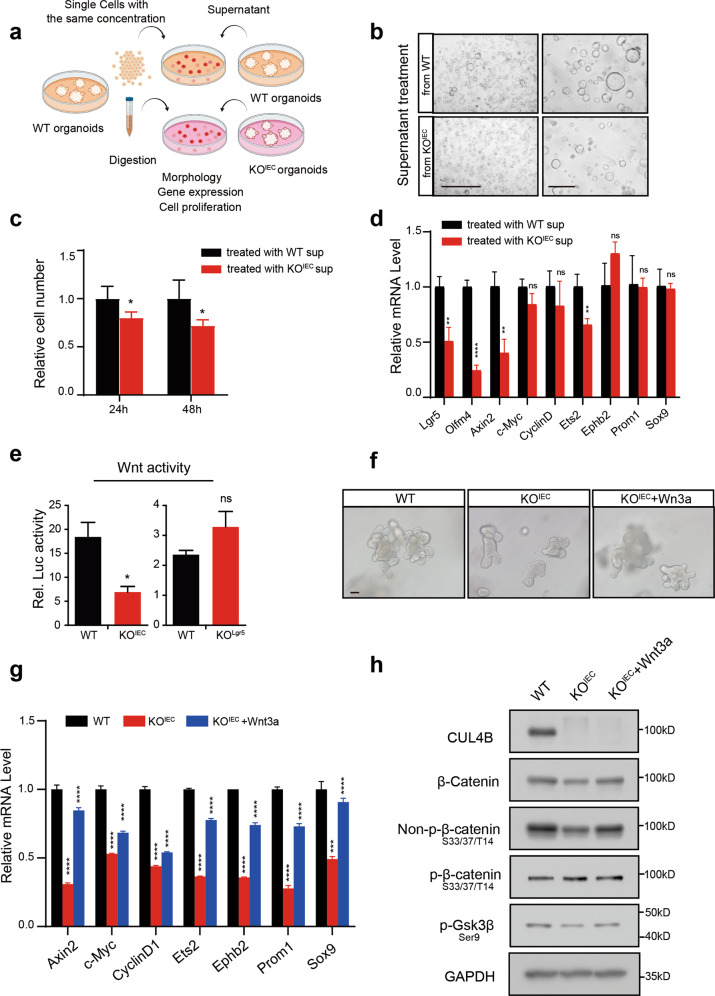
Decreased self-renewal and budding efficiency in CUL4B-deficient organoids are rescued by Wnt3. **a** Illustration of supernatant treating assay. The same number of wildtype SI organoids treated with supernatant collected from WT or KO^IEC^ organoids with the same cell number for 24 or 48 h. **b**, **c** The representative images (**b**) and cell numbers (**c**) of wild type small intestine organoids treated with supernatant collected from WT or KO^IEC^ organoids with the same cell number cultured for 24 or 48 h. The scale bar is 1000 μm (Left), 400 μm (Right). *P < 0.05. **d** The mRNA level of stem cell markers and Wnt target genes in wildtype SI organoids treated with supernatant collected from WT or KO^IEC^ organoids. The experiment was performed with four replicates. **P < 0.01, ****P < 0.0001, ns, no significance. **e** The relative luciferase activity of Wnt reporter cells were detected after treatment with supernatant collected from KO^IEC^ and KO^Lgr5^ and WT organoids for 48 h. The same cell number of organoids at the third passage were cultured and seeded. *P < 0.01, ns, no significance. **f** The representative images of SI organoids from WT and KO^IEC^ mice with or without treatment with recombinant Wnt3a (100 ng/ml). The scale bar is 50 μm. **g**, **h** The relative mRNA levels of Wnt/β-catenin target genes and protein level of Wnt pathway in WT and KO^IEC^ SI organoids with or without treatment with recombinant Wnt3a (100 ng/ml). ***P < 0.001, ****P < 0.0001. Error bars represent standard errors in the figure.

To determine the potential roles of CUL4B in regulating ISC niche, we first examined the effect of depletion of CUL4B in Lgr5^+^ cells by treating KO^Lgr5^ organoids with tamoxifen. With 24 h transient induction, knockout of *Cul4b* only decreased cell proliferation but did not alter organoid-forming and budding efficiency, implying an indispensable role of CUL4B in non-stem cells (Supplementary Fig. S4a–c). To substantiate this notion, we prepared conditional medium from cultured WT and KO^IEC^ organoid and assessed the effect of CUL4B deletion on their secretory phenotypes by growing single cells of wildtype organoids in the conditional medium (illustrated in Fig. 5a). Compared to the organoids cultured in WT conditional medium, the organoids cultured in KO^IEC^ supernatant grew much more slowly (Fig. 5b, c). qRT-PCR confirmed the down-regulation of Wnt target genes in organoids treated by KO^IEC^ supernatant (Fig. 5d). Furthermore, a Wnt luciferase reporter system was used to detect Wnt activity. The reporter cells treated with supernatant from KO^IEC^ organoids, but not supernatant from KO^Lgr5^ organoids revealed a significantly reduced Wnt activity (Fig. 5e). These data suggest that the function of CUL4B in non-stem cells is also essential to maintain ISC function. Consistently, the addition of recombinant Wnt3a rescued the reduction of budding formation and Wnt pathway in KO^IEC^ organoids (Fig. 5f–h).

### *Cul4b* deficiency impairs Paneth cell development

CUL4B maily expressed at crypt bottom including ISCs and Paneth cells, which located among ISCs and provided the main cell source of secreted Wnt proteins. As the role of CUL4B in non-stem cells is indispensable, we next examined if the Paneth cells were altered by CUL4B deletion. Consistant with the observation in lysosome staining of small intestine tissues, we observed a significant reduction of Paneth cells per bud in KO^IEC^ organoids. In contrast, increased number of Paneth cells per bud was detected in *CUL4B* transgenic organoids (Supplementary Fig. S5a, b). UEA-1 staining confirmed that depletion of CUL4B from epithelial cells led to the loss of Paneth cells (Supplementary Fig. S5c). Furthemore, the impaired structure of Paneth cells was confirmed by Transmission Electron Microscope (TEM). Abnormal cell morphology including degranulation, disturbed cell junction, increased lysosome and disordered mitochondrion were found in KO^IEC^ intestines (Supplementary Fig. S5d). These data indicate that lack of CUL4B in intestine epithelial leads to abnormal Paneth cell number, inner and inter cell structure.

### CRL4B targets intestinal IRGM1 for degradation

**Fig. 6:**
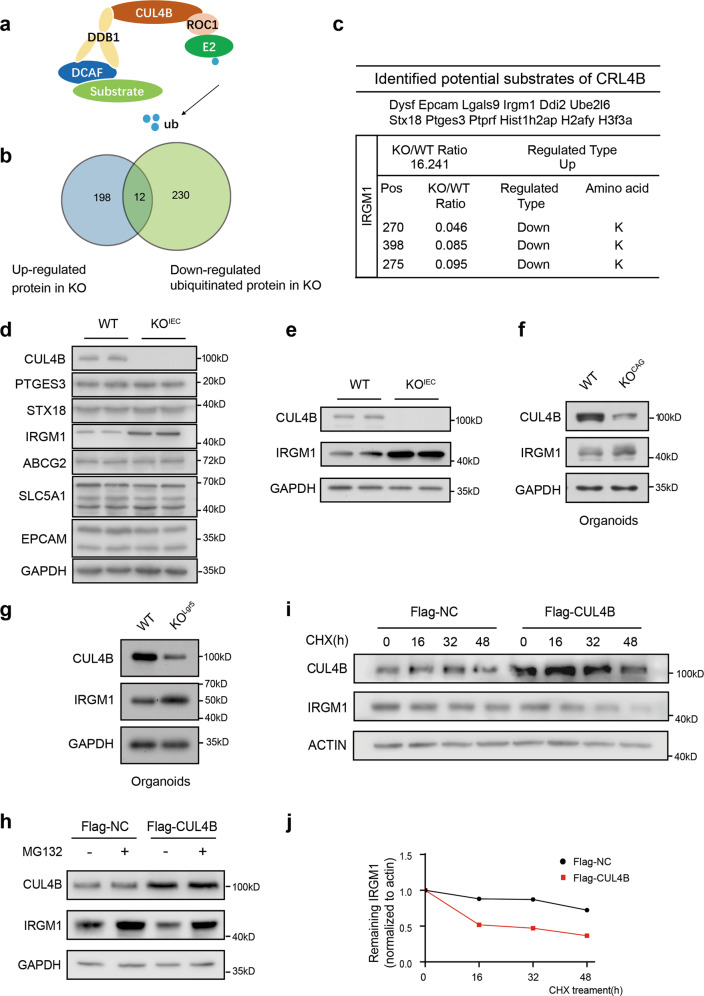
CRL4B functions as the E3 ligase to ubiquinate IRGM1. **a** The structure of CRL4B E3 ligase. **b** Venn diagram showing the overlap of up-regulated proteins and down-regulated ubiquitylated proteins identified in KO^IEC^ crypts by Mass Spectrometry (MS). **c** The name list of twelve overlapping proteins. The changing folds and detail modification sites of IRGM1 were indicated. **d** Western blots analysis of the overlapping proteins identified by proteomics. **e**–**g** Western blots were performed to check IRGM1 accumulation in KO^IEC^ crypts (**e**) KO^CAG^ organoids (**f**) and KO^Lgr5^ organoids (**g**) compared to WT. CUL4B, IRGM1, and GAPDH expression were detected. **h** The IRGM1 level was detected in CUL4B overexpression (Flag-CUL4B) and control (Flag-NC) 293 T cells by Western blot with or without the presence of proteasome inhibitor MG132. The cell lysate was then detected with indicated antibodies. **i, j** The effect of CUL4B on the IRGM1 stability was detected by Western blot in CUL4B overexpression (Flag-CUL4B) and control (Flag-NC) 293 T cells with CHX chasing. The cell lysate was then detected with indicated antibodies with ACTIN as the control (**i**). **j** Signals from immunoblots were analyzed by Quantity One (Bio-Rad) to indicate the half-life of IRGM1 degradation.

CUL4B, as a core component of an E3 ubiquitin ligase complex, associates with DDB1, ROC1, DDB1-and CUL4-associated factor (DCAF) to degrade substrates by 26 S proteasome (Fig. 6a). To reveal the underlying molecular mechanism, we performed proteomics to identify the altered proteins and ubiquitinated proteins in *Cul4b*-deficient crypts. 210 upregulated and 293 downregulated proteins were identified in *Cul4b*-deficient mice with the fold change above 1.5 (Supplementary Table S3). The ubiquitinated levels of 251 lysines of 180 proteins were upregulated and 321 lysines of 242 proteins were downregulated with more than 1.5 folds (Supplementary Table S4). Venn analysis of candidate degradation substrates of CUL4B was among the overlapping proteins with upregulated expression and reduced ubiquitinated level (Fig. 6b). Twelve potential substrates were identified, among which immunity-related GTPase subfamily protein (IRGM1) had the biggest change at protein level and most decreased ubiquitinated sites (Fig. 6c). We then performed immunoblot to confirm the expression change of these candidate substrates (Fig. 6d). IRGM1 was significantly upregulated both in isolated crypts and cultured organoids derived from KO^IEC^ and KO^Lgr5^ mice (Fig. 6e-g). Meanwhile, overexpression of CUL4B resulted in a reduction of IRGM1 protein. Importantly, the reduction of IRGM1 caused by the overexpression of CUL4B was efficiently blocked by the administration of MG132, a proteasome inhibitor (Fig. 6h). The results imply that CUL4B degraded IRGM1 via a proteasome-dependent degradation mechanism. To further strengthen this notion, we measured the half-life of IRGM1 protein in CUL4B overexpressed cells. Remarkably, overexpression of CUL4B resulted in a accelarated IRGM1 decay (Fig. 6i, j). These results suggest that CUL4B decreases the stability of IRGM1 protein.

### CRL4B functions as an E3 ligase for intestinal IRGM1 at Lys270

**Fig. 7:**
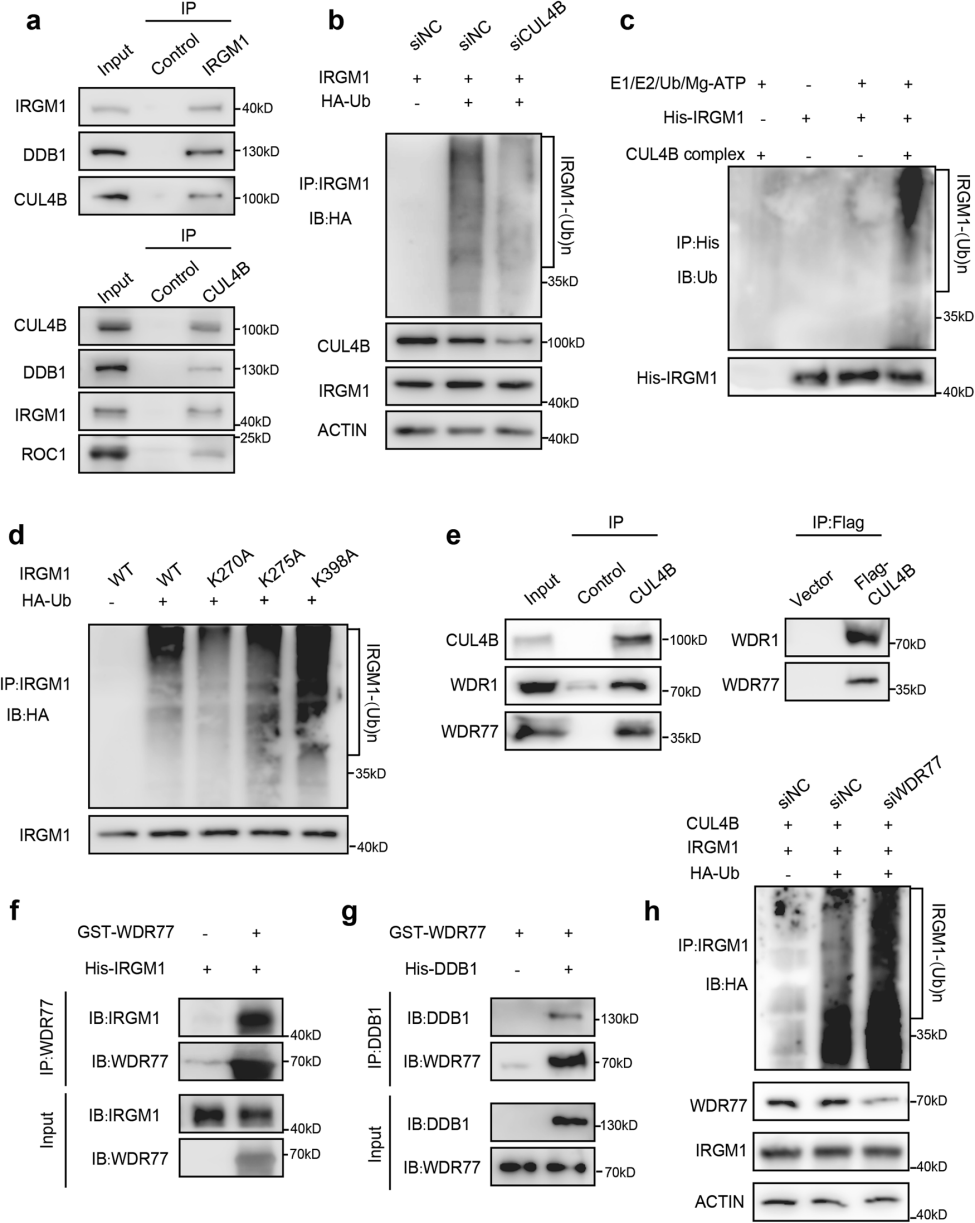
IRGM1 is polyubiquitylated and degraded by CRL4B complex. **a** Immunoprecipitation was performed to detect interactions between CUL4B, DDB1 and IRGM1. Protein extracted from small intestine of mice was immunoprecipitated with the indicated antibodies individually. **b** The effect of CUL4B on IRGM1 ubiquitination was confirmed by immunoprecipitation and detected by Western blot with indicated antibodies. IRGM1, HA-Ub expression constructions and siRNA (siCUL4B or siNC) were transfected into HEK293T cells as indicated in lane 1, 2, and 3. **c** In vitro ubiquitination of IRGM1 by the CRL4B complex. Purified His-IRGM1 was incubated with E1, E2, Ub and ATP in the absence and presence of the CRL4B complex (Flag-CUL4B IP products) as indicated in lane 1–4. Ubiquitination of His-IRGM1 was analyzed by Western blot using anti-Ub antibody. **d** Single lysine mutations on IRGM1 defect ubiquitination by CRL4B. Immunoprecipitation was performed with wildtype and mutant *Irgm1* variants together with Flag-CUL4B, HA-Ub with treatment with MG132 for 3 h as indicated in lane 1–5. Western blot was performed and detected with indicated antibodies. Input (3%) was used for Western blotting. **e** Immunoprecipitation was performed to detect interactions between CUL4B and WDR77. Protein extracted from mice small intestine or HEK293T cells was immunoprecipitated with the indicated antibodies. **f**, **g** GST pull down assays identify the direct interaction among IRGM1, DDB1 and WDR77. GST-WDR77, His-DDB1 and His-IRGM1 were expressed in *E. coli* individually followed by protein purification and immunoprecipitated pull-down. The proteins were detected using indicated antibodies. Input (15%) was used for Western blot. **h** In vivo ubiquitination assay of IRGM1 by the CRL4B complex with or without WDR77 interfering.

We then tested whether IRGM1 was a direct substrate of CRL4B. Firstly, we confirmed the physical association between CRL4B complex and IRGM1. IRGM1 was immunoprecipitated with a substantial amount of CUL4B and DDB1. IRGM1 was coimmunoprecipitated with antibodies against CUL4B (Fig. 7a). The ubiquitination assay showed CRL4B significantly increased the amount of polyubiqutinated IRGM1, whereas knockdown of *CUL4B* resulted in a significant reduction (Fig. 7b, c) FLAG-CUL4B immunoprecipitated complex was confirmed in Supplementary Fig. S6a. These results support CRL4B as an E3 ligase for IRGM1. Residues Lys270, Lys275 and Lys398 on IRGM1 protein were identified as potential ubiquitination sites by quantitative mass spectrometry. To examine whether CRL4B targets these residues, we mutated Lys270/275/398 to alanine respectively and performed the ubiquitination assay. Our results showed that K270A, but not K275A or K398A mutant significantly reduced the level of polyubiquitinated IRGM1, suggesting that Lys270 was targeted by CRL4B complex (Fig. 7d).

CRL4B uses a variety of DCAFs to assemble different E3 ligases to specifically target substrates. Mass spectrometry identified several CUL4B-interacting proteins including WD40 containing proteins such as WDR1, WDR3, WDR18 and WDR77, among which WDR77 was physically associated with CUL4B, DDB1 and ROC1, as demonstrated by coimmunoprecipitation (Fig. 7e). Importantly, WDR77 was co-expressed with CUL4B at intestinal crypts (Supplementary Fig. S6b). GST-pulldown assay confirmed the direct interaction between WDR77 and IRGM1 as well as DDB1 (Fig. 7f, g). These data suggest that WDR77 functions as a substrate receptor, directly recruits substrate IRGM1, facilitates its ubiquitination, and promotes subsequent proteasomal degradation. However, interfering *WDR77* did not lead to significant decrease of ubiquitinated IRGM1 (Fig. 7h). Taken together, our results suggest CRL4B as an E3 ligase for intestinal IRGM1 at Lys270.

### CUL4B regulates Paneth cell function and organoid proliferation through IRGM1

**Fig. 8:**
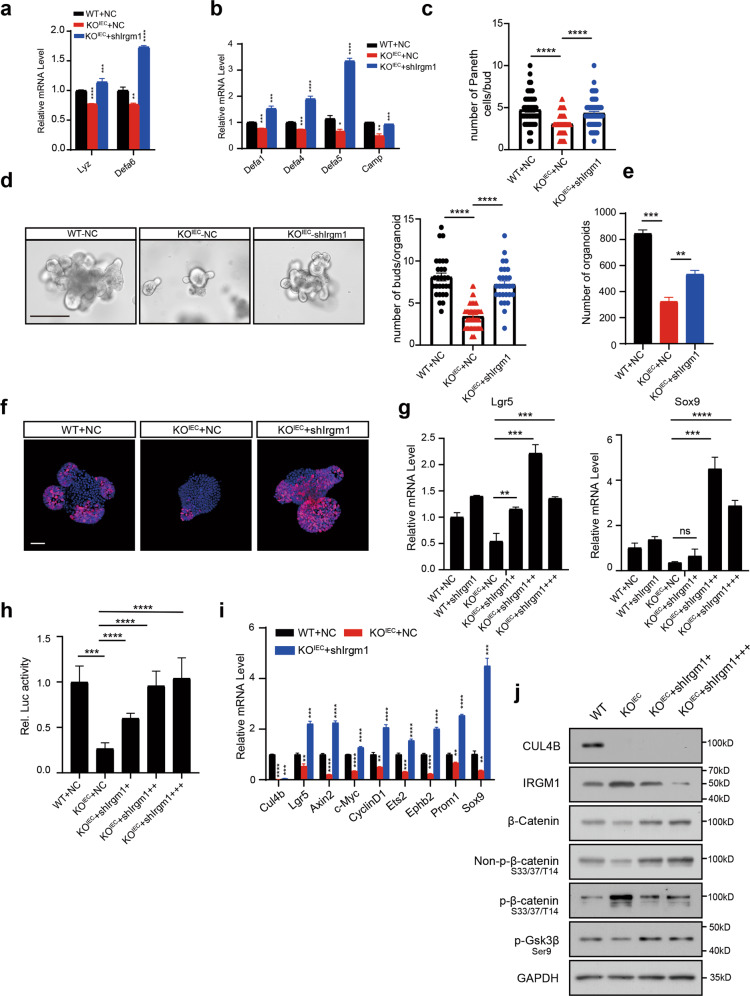
IRGM1 mediates the impaired proliferation and Paneth cell function caused by Cul4b deletion. **a, b** Relative mRNA levels of antimicrobial peptides cryptdin (Defa1), Defa4, Defa5, cathelicidin (Camp), Defa6, and lysozyme (Lyz) in WT + NC, KO^IEC^ infected by lentivirus to knockdown *Irgm1* (KO^IEC^ + shIrgm1) and KO^IEC^ control organoids (KO^IEC^ + NC). *P < 0.05, **P < 0.01, ***P < 0.001, ****P < 0.0001. **c**. The average number of Paneth cells within individual buds (right) from WT + NC, KO^IEC^ + shIrgm1 and KO^IEC^ + NC. ****P < 0.0001. **d** Representative image the organoids (left) of WT (WT + NC), KO^IEC^ infected by lentivirus to knockdown *Irgm1* (KO^IEC^ + shIrgm1) and control (KO^IEC^ + NC). The average budding number per organoid (right) from WT + NC, KO^IEC^ + shIrgm1 and KO^IEC^ + NC. The scale bar is 200 μm. ****P < 0.0001. **e** The organoids numbers of WT (WT + NC), KO^IEC^ infected by lentivirus to knockdown *Irgm1* (KO^IEC^ + shIrgm1) and control (KO^IEC^ + NC). **P < 0.01, ***P < 0.001. **f** Representative image of Ki67 staining from organoids of WT (WT + NC), KO^IEC^ infected by lentivirus to knockdown *Irgm1* (KO^IEC^ + shIrgm1) and control (KO^IEC^ + NC). The scale bar is 15 μm. **g** Relative mRNA levels of stem cell marker *Lgr5* (Left) and *Sox9* (Right) in organoids of WT + NC, KO^IEC^ infected by dose-dependent lentivirus to knockdown *Irgm1* (KO^IEC^ + shIrgm1) and control (KO^IEC^ + NC). The experiment was performed with triplicates. **P < 0.01, ***P < 0.001, ****P < 0.0001, ns, no significance. **h** Relative luciferase activity of HEK293 STF Wnt activity reporter lines were detected after 48 h treated with secreted supernatant collected from organoids WT + NC, KO^IEC^ infected by dose-dependent lentivirus to knockdown *Irgm1* (KO^IEC^ + shIrgm1) and control (KO^IEC^ + NC). ***P < 0.001, ****P < 0.0001. **i** Relative mRNA levels of Wnt target gene in organoids of WT (WT + NC), KO^IEC^ infected by lentivirus to knockdown *Irgm1* (KO^IEC^ + shIrgm1) and control (KO^IEC^ + NC). The experiment was performed with triplicates. **P < 0.01, ***P < 0.001, ****P < 0.0001, ns, no significance. **j** Protein level of Wnt/β-catenin pathway was determined by Western blot in SI organoids from KO^IEC^, KO^IEC^ with *Irgm1* ablation (KO^IEC^ + shIrgm1) and WT (WT + NC) mice.

IRGM1 is the autophagy-associated protein susceptible for intestinal inflammation and microbiology infection. *Irgm1* KO mice display granules in Paneth cell being finer, irregular in size, and less dense in appearance, selective reduction of Lyz expression and altered antimicrobial peptide (AMP) production [29–31]. To check whether CUL4B regulate Paneth cell structure and function through IRGM1, we knocked down *Irgm1* by lentivirus in organoids from KO^IEC^ mice to check AMP expression. The reduced number of Paneth cells and transcript levels of lysosome and AMPs in *Cul4b* KO^IEC^ organoids was rescued by deletion of *Irgm1* (Fig. 8a, b). Decreased organoid formation, budding efficiency and cell numbers per bud were rescued after deletion of *Irgm1* as well (Fig. 8c–e). Accordingly, proliferation capacity of organoids was also elevated in *Cul4b* KO^IEC^ organoids by *Irgm1* deletion (Fig. 8f). Taken together, our findings suggest that decreased Paneth cell number, morphology and function by lack of CUL4B was mediated through IRGM1 accumulation.

### Dysregulated Wnt signaling caused by CUL4B depletion is rescued by knockdown of *Irgm1*

To test whether the dysregulation of Wnt signaling in *Cul4b*-deficient organoids was caused by increased levels of IRGM1, we examined the rescue effect of *Irgm1* knockdown on the Wnt activity and its target genes in *Cul4b* KO^IEC^ organoids. Infection of *Irgm1* knockdown lentivirus significantly upregulated the transcription of Wnt target genes (*Lgr5* and *Sox9*) and Wnt luciferase activity in a dose-dependent manner (Fig. 8g, h). Interfering *Irgm1* efficiently attenuated the reduction of other Wnt target genes and proteins in *Cul4b* KO^IEC^ organoids (Fig. 8i, j). Overall, our findings demonstrated a critical role of CUL4B in intestinal development and homeostasis. Mechanistically, we identified IRGM1 as a novel targeting substrate of CRL4B complex. CUL4B ubiquitylates IRGM1 at K270. Impaired intestinal homeostasis caused by CUL4B deletion is mediated by upregulation of IRGM1.

## Discussion

In this study, we have demonstrated a critical role of CUL4B in intestinal homeostasis. We observed that ISC self-renewal and differetiation were impaired in the absence of CUL4B. Mechanistically, CUL4B regulates ISCs and their niches by targeting IRGM1 for ubiquitination and degradation, and thereby sustaining Wnt signaling. Our results have important implications in understanding the regulatory network of intestine development and related diseases.

Due to lack of Lgr5^+^ ISCs, CUL4B KO^IEC^ intestine shows a significant reduction in self-renewal. Constitutive gradient Wnt enriched at crypts was required for ISC proliferation and specific lineage differentiation [7, 32, 33]. The dysregulated Wnt signaling in *Cul4b* KO^IEC^ mice not only causes shorter crypts/villi length, but also leads to loss of secretory lineage differentiation including Paneth cells, enteroendocrine cells and goblet cells. In contrary, the function of absorptive lineage of enterocytes is accentuated in KO^IEC^ intestine with upregulated *Alpi* staining and *Apoa4* gene expression. The RNA-seq data show that the type 2 diabetes, PPAR pathway and insulin signaling are enriched in KO^IEC^ tissue, which is in accordance with our previous reports that adipocyte specific *Cul4b* knockout mice are susceptible to obesity [25, 27]. Our results further indicate CUL4B deficiency as a risk factor for metobolic disorders.

Through secreting Wnt3, EGF and Notch that fuel stem cell self-renewal, Paneth cells play critical roles in ISC maintenance as one of the major components of ISC niches [2, 34, 35]. The reduction of organoid-forming and budding efficiency following CUL4B deletion is restricted to ex-vivo cultured organoids derived from KO^IEC^ rather than KO^Lgr5^ intestine. The transient knockout of CUL4B with short-term tamoxifen induction in KO^Lgr5^ organoids leads to reduced size. But in single-cell assay, the expansion medium may contain enough Wnt signals that rescue the decreased organoid formation in KO^Lgr5^ organoids.

Besides, our results of supernatant collection clearly demonstrate that deletion of CUL4B influences ISC stemness by extracellular environment through secretion. The TEM confirms the dysregulated intercellular structure and cell-cell junction between Paneth cell and ISCs in KO^IEC^ mice, that also has been proved as a cause of disrupted Wnt recognization [7, 32]. IFN-gamma, which has been reported to trigger degranulation and extrusion of Paneth cells and release of antimicrobial products, is also enriched in CUL4B KO^IEC^ tissues (data not shown). Taken together, our fingdings suggest the essential role of CUL4B in ISC niche. Increased numbers of granulated Lyz^+^ cells has been observed in *Irgm1* KO^IEC^ mice [31]. Consistantly, the Lyz^+^ cells were significantly reduced following *Cul4b* deletion. We perform dose-dependent lentivirus infection of sh*Irgm1* in KO^*IEC*^ organoids to clarify that CUL4B regulates Wnt regulation through IRGM1. Total and active β-catenin are rescued in KO^IEC^ organoids after *Irgm1* deletion and Wnt recovery is confirmed by the STF reporter system. IRGM1 ablation also has strong rescue effect to re-produce antimicrobial products of Defa1 Defa4, and Defa6 in KO^IEC^ organoids. Collectively, these data strongly suggest a non-redundant role of CUL4B in Paneth cell partially by degrading IRGM1. Besides, IRGM1 has been linked to bacterial immunity, inflammation, and autophagy initiation complexes formation by co-assembling with ULK1 and Beclin1 [15, 29]. Human IRGM1 is a risk allele in Crohn’s disease (CD), one chronic, immune-mediated, inflammatory intestinal disorder. Whether CUL4B functions in host-defense and chronic intestinal inflammation will be determined in our future study.

Our results reveal CRL4B as an E3 ubiquitin ligase to ubiquitylate IRGM1 at K270 and consequently degrade IRGM1. Several candidate WD40 repeat proteins are found by mass spectrometry as potential DCAFs for IRGM1. Although we identify a direct association between WDR77 with DDB1, interfering *WDR77* by siRNA in 293 T cells results in no IRGM1 accumulation. In vitro ubiquitylation assay also suggests WDR77 is dispensable for IRGM1 degradation. The other co-factors of CRL4B complex for IRGM1 ubiquitylation need to be further explored.

CUL4B is mainly expressed in the cytoplasm of ISCs zone, which is recapitulated in ex vivo intestinal organoids. Although CUL4B is characterized by the nuclear localization signals at the N terminus, we uncover the specific intestinal cytoplasm location of CUL4B. Our results suggest the cytoplasm location of CUL4B could not be changed in organoids treated with nuclear export inhibitor, Leptomycin B (data not shown). Further experiments are needed to elucidate the mechanism by which CUL4B is excluded from the nucleus in the intestine.

Overall, our findings demonstrate a critical role of CUL4B in intestinal development and homeostasis. A cytoplasm specific expression of CUL4B is found at bottom crypts. Deletion of *Cul4b* leads to reduced self-renewal and an impaired lineage differentiation towards secretory progenitors of ISCs through Wnt signals. Mechanistically, CRL4B complex ubiquitylate its substrate IRGM1 at K270. These findings advance our understanding of how intestinal homeostasis is regulated and have potential implications in the management of gasteroenterological diseases.

## Supplementary information

Supplementary_Figures and Figure Legends

Change of Authorship Request

Supplementary materials for WB

Table S1 The list of primers used for qRT-PCR.

Table S2 The differential genes changed in KOIEC mice (N=4), in KOCAG organoids (N=2) or in KOIEC organoids (N=2) compared with wildtype intestine.

Table S3 The differential proteins changed in KOIEC mice with fold change of above 1.5 compared with wildtype.

Table S4 The differential ubiquitylated proteins changed in KO<IEC> mice with fold change of above 1.5 compared with wildtype.

Table S5 The reagents and resource we used in the manuscripts.

Movie S1 Co-staining of CUL4B and β-catenin in small intestine organoids.

Movie S2 The differential ubiquitylated proteins changed in KOIEC mice with fold change of above 1.5 compared with wildtype.

Movie S3 z-stack re-construction of Ki67 staining in KOIEC small intestine organoids

Reproducibility Checklist

## Data Availability

Raw and processed data for RNA-seq can be reached from GEO database with the number of GSE157818 and MS data in PRIDE with the number of PXD-021528. All other datasets and code are available upon request from the huhuili@sdu.edu.cn.
