# Supplementary material for: Cullin 4b-RING ubiquitin ligase targets IRGM1 to regulate Wnt signaling and intestinal homeostasis
Source: Cell Death Differ. 2022 Feb 23;29(9):1673–88. doi: 10.1038/s41418-022-00954-9 (PMC9433385; doi:10.1038/s41418-022-00954-9)
Supplement: Supplementary file 1 — Supplementary_Figures and Figure Legends [file 41418_2022_954_MOESM1_ESM.docx]

Supporting Information

Title Cullin 4b-RING ubiquitin ligase targets IRGM1 to regulate Wnt signaling and intestinal homeostasis

Yujia Fan^1^, Xiaohan Huo^1^, Beibei Guo^1^, Xiaohui Zhang^1^, Yang Yang^1^, Jiabei Lian^1^, Xinyuan Meng^1^, Yiwen Shao ^1^, Yongxin Zou^1^, Haiyang Guo^2^, Haitao Wang^3^, Gongping Sun^4^, Hao Dou^1^, Jinshen Wang^6^, Changshun Shao^5^, Yaoqin Gong^1, *^, Huili Hu^1, *^


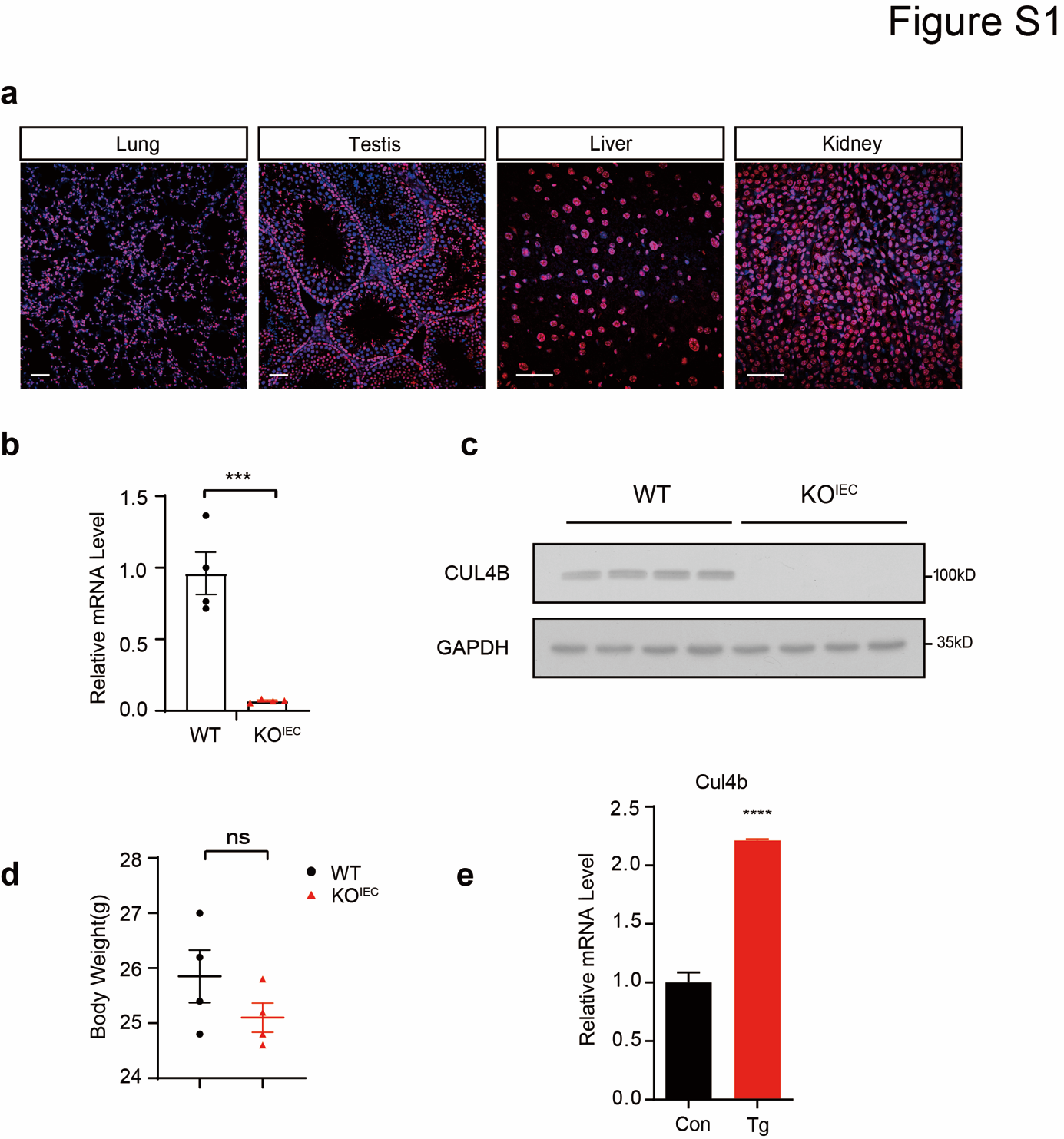


**Fig. S1: The expression level of CUL4B deletion and overexpression was confirmed.**

**a** Immunofluorescent staining of CUL4B in murine lung, testis, liver and kidney. Red, CUL4B, Blue, DAPI. The scale bar is 50 μm. **b** The mRNA level of *Cul4b* in the crypts isolated from KO^IEC^ mice relative to WT littermates. ****P* <0.001. **c** Western blots analysis of CUL4B expression in the crypts from KO^IEC^ mice and WT littermates. **d** No significance of body weight was found between WT and KO^IEC^ mice. ns, no significance. **e** The mRNA level of *CUL4B* in the crypts from transgenic mice (Tg) relative to control littermates (Con). *****P* <0.0001.


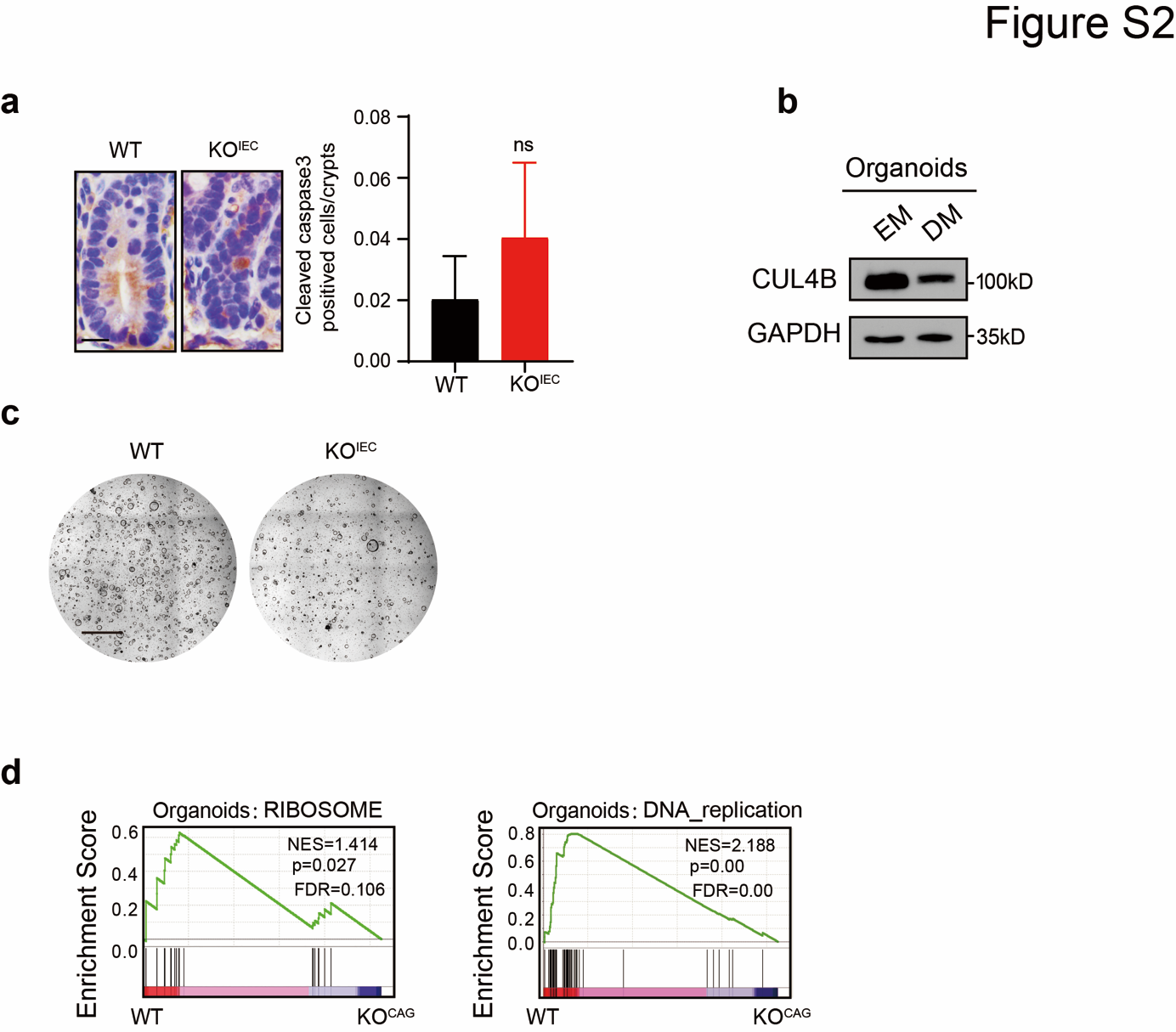


**Fig. S2: Cell proliferation instead of apoptosis was responsible for decreased self-renewal after CUL4B deletion.**

**a** The representative Images and quantification of cleaved Caspase3 staining to detect apoptosis in intestine of KO^IEC^ and WT mice. The scale bar is 50 μm. ns, no significance. **b** Western blots show the expression level of CUL4B in intestine organoids cultured in stem cell-enriched expansion medium (EM) or differentiation medium (DM). **c** The representative images of organoids starting from single cells in EM derived from KO^IEC^ and WT mice. The scale bar is 1000 μm. **d** GSEA analysis for differentiated expression genes of organoids between KO^CAG^ and WT mice. Ribosome and DNA replication pathways were enrichment in WT group. NES, normalized enrichment score; FDR, false discovery rate.


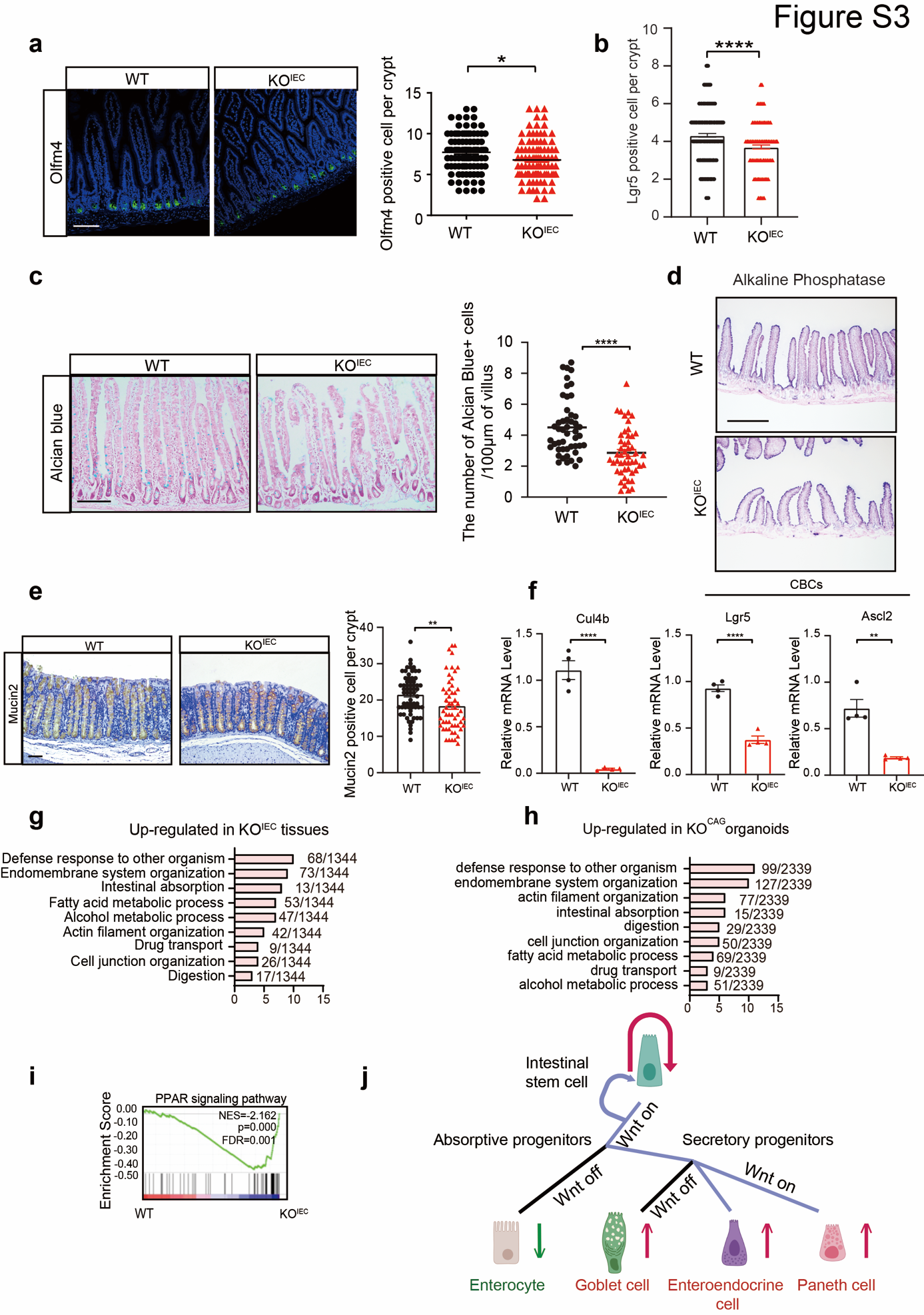


**Fig. S3: Transcriptional alterations in KO^IEC^ intestines.**

**a** The representative images and quantification of Olfm4 staining in SI of KO^IEC^ and WT mice (N=3). The scale bar is 50 μm. **P <0.05.* **b** Fluorescence FISH analysis of ISC marker Lgr5 in KO^IEC^ and WT crypts (*N*= 3). **c** The representative images and quantification of Alcian blue staining in SI of KO^IEC^ and WT mice (*N*=3). The scale bar is 50 μm. *****P* <0.0001. **d** The representative images of Alkaline Phosphatase staining in SIs of KO^IEC^ and WT mice. The scale bar is 50 μm. **e** The representative images and quantification of Mucin2 staining in colon of KO^IEC^ and WT mice (*N*=3). The scale bar is 50μm. ***P* <0.01. **f** The mRNA expression level of stem cell markers in KO^IEC^ colon relative to WT littermates. ***P* <0.01, *****P* <0.0001. **g** GO pathway analysis was performed to indicate significantly differentiated up-regulated pathways in KO^IEC^ intestine. **h** GO pathway analysis was performed to indicate significantly differentiated up-regulated pathways in KO^IEC^ organoids. **i** PPAR pathway was enriched in KO^IEC^ intestine. **j** Graphic abstract of major signals CUL4B affected in ISC self-renewal and differentiation. Red, ISC self-renewal and differentiation CUL4B promoted; Green, differentiation CUL4B repressed.


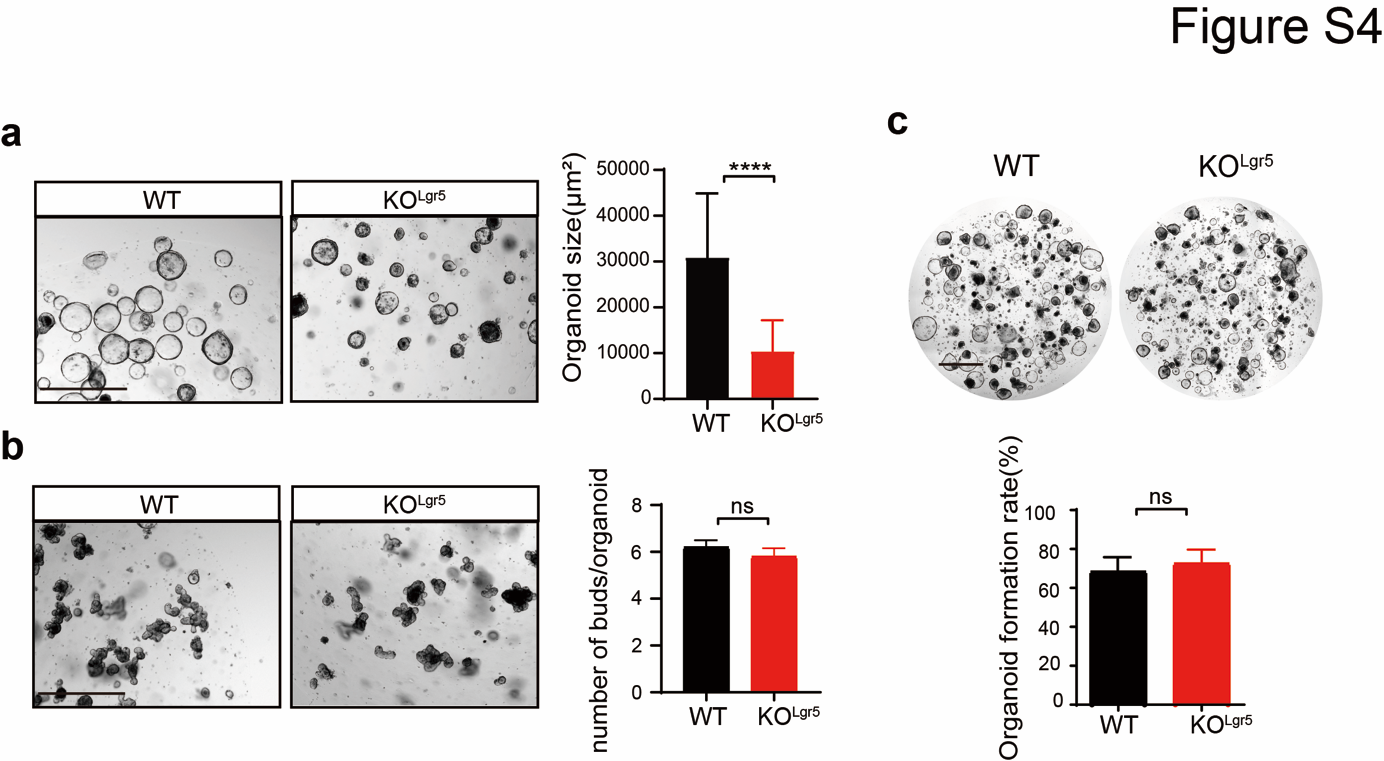


**Fig. S4: Transient CUL4B deletion in Lgr5^+^ cells reduced organoid size instead of formation efficacy**

**a** The representative images (left) of small intestine (SI) organoids cultured in expansion medium from KO^Lgr5^ mice and WT mice. The scale bar is 1000 μm. Quantification(right) of SI cystic cross-sectional area of organoids. Data are representative of three independent experiments. *****P*<0.0001. **b** The average budding number per organoid from WT and KO^Lgr5^ mice. ns, no significance. **c** Colony-forming assays were performed to determine the cell viability of organoids cultured in EM from KO^Lgr5^ and WT mice. The number of colonies per well was counted. The scale bar is 1000mm. *****P*<0.0001.


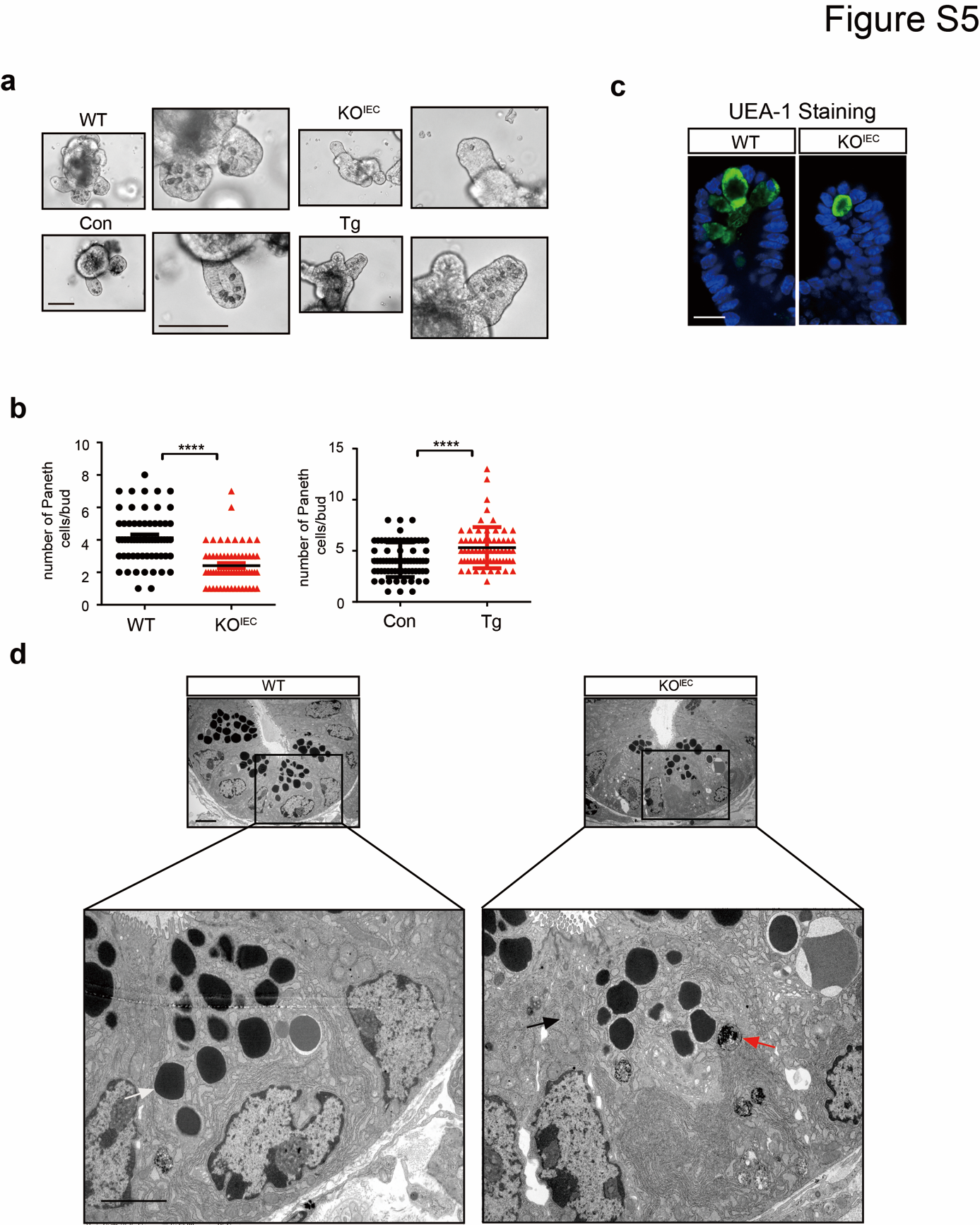


**Fig. S5:** **CUL4B deletion led to reduced number and dysregulated cellular structure of Paneth cells**.

**a** The representative images of Paneth cells identified as granular cells using phase contrast microscopy within individual buds from WT/KO^IEC^ or Tg/Con mice. The scale bar is 50μm (Left), 100μm (Right). **b** The average number of Paneth cells within individual buds from WT/KO^IEC^ or Con/Tg mice are shown. *****P*<0.0001. **c**) Whole-mount analysis of UEA-1(green) in organoids from KO^IEC^ and WT mice. Blue, DAPI. The scale bar is 15μm, *****P*<0.0001. **d** Transmission electron microscopy analysis of the crypts of KO^IEC^ and WT mice. The white arrow marks PC granules; The black arrow marks mitochondria; Lysosome are marked with a red arrow. The scale bar is 5μm (Upper), 2μm (Lower).


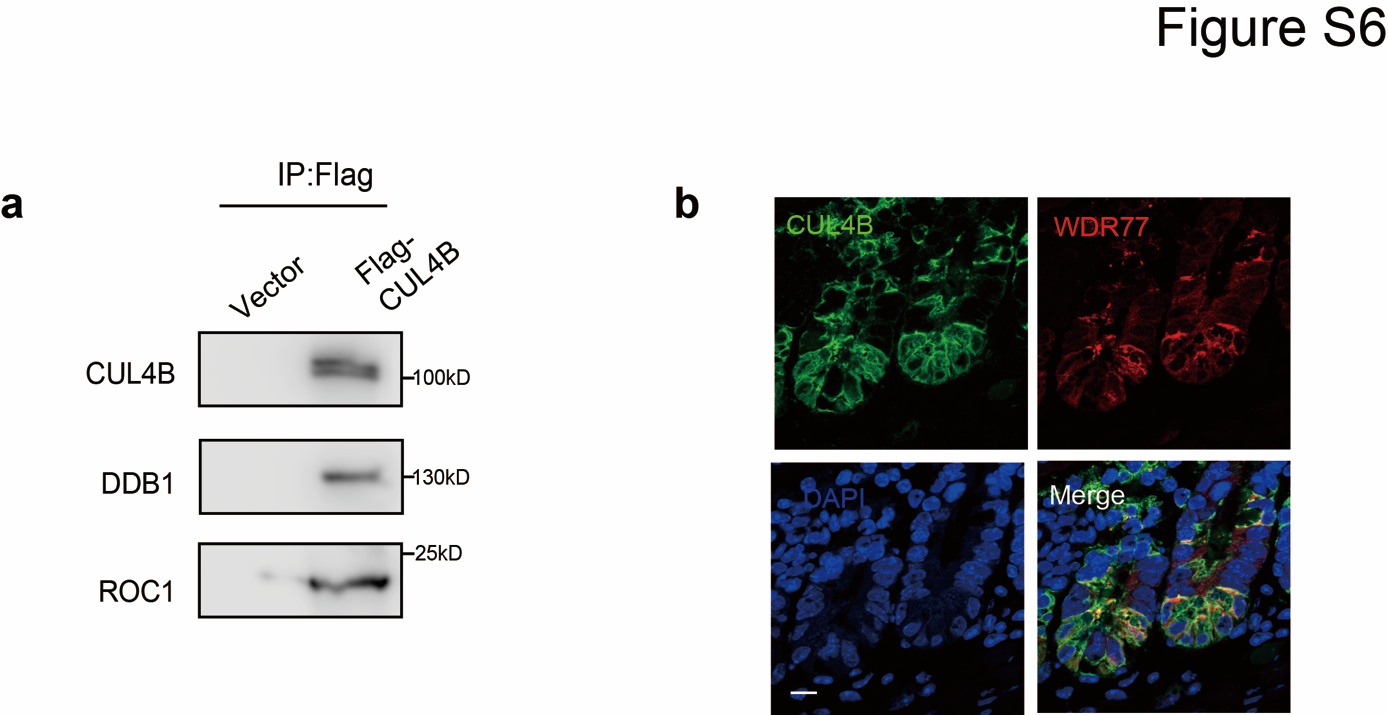


**Fig. S6: CRL4B associates with WDR77 to degrade IRGM1**

**a** Immunoprecipitation was performed to detect interactions between CUL4B, DDB1 and ROC1. Protein extracted from CUL4B overexpression (Flag-CUL4B) and control 293T cells. **b** Co-staining of WDR77 and CUL4B in small intestines. Red, WDR77, Green, CUL4B, Blue, DAPI. The scale bar is 50 μm.

**Table S1**

The list of primers used for qRT-PCR.

**Table S2**

The differential genes changed in KO^IEC^ mice (*N*=4), in KO^CAG^ organoids (*N*=2) or in KO^IEC^ organoids (*N*=2) compared with wildtype intestine.

**Table S3**

The differential proteins changed in KO^IEC^ mice with fold change of above 1.5 compared with wildtype.

**Table S4**

The differential ubiquitylated proteins changed in KO^IEC^ mice with fold change of above 1.5 compared with wildtype.

**Table S5**

The reagents and resource we used in the manuscripts.

**Movie S1**

Co-staining of CUL4B and β-catenin in small intestine organoids.

**Movie S2**

z-stack re-construction of Ki67 staining in wildtype small intestine organoids.

**Movie S3**

z-stack re-construction of Ki67 staining in KO^IEC^ small intestine organoids.

**Supplementary materials for WB**

All data of western blots for the manuscript
