# Supplementary material for: Cullin 4b-RING ubiquitin ligase targets IRGM1 to regulate Wnt signaling and intestinal homeostasis
Source: Cell Death Differ. 2022 Feb 23;29(9):1673–88. doi: 10.1038/s41418-022-00954-9 (PMC9433385; doi:10.1038/s41418-022-00954-9)
Supplement: Supplementary file 2 — Change of Authorship Request [file 41418_2022_954_MOESM2_ESM.pdf]

SPRINGER NATURE

Change of authorship request form - Journals (pre-acceptance)

Important information. Please read.

- This form should be used by authors to request any change in authorship (adding/deleting authors) including changes in corresponding authors. This form should not be used for name changes. Please fully complete all sections. Use black ink and block capitals and provide each author's full name with the given name first followed by the family name.

- By signing this declaration, all authors guarantee that the order of the authors are in accordance with their scientific contribution, if applicable as different conventions apply per discipline, and that only authors have been added who made a meaningful contribution to the work.

- Please note, in author collaborations where there is formal agreement for representing the collaboration, it is sufficient for the representative or legal guarantor (usually the corresponding author) to complete and sign the Authorship Change Form on behalf of all authors, **next to the added/removed author(s)**. **(Complete Section 3, followed by Section 6.)**
  In author collaborations where there is no formal agreement for representing the collaboration and **there are more than 10 authors**, one may sign for all, provided the signer appends correspondence that attests that each of the authors have agreed to the change **and the added/removed authors sign the form**. **(Complete Section 3, followed by Section 6.)**

- Please note, we cannot investigate or mediate any authorship disputes. If you are unable to obtain agreement from all authors (including those who you wish to be removed) you must refer the matter to your institution(s) for investigation. Please inform us if you need to do this.

- If you are not able to return a fully completed form within **30 days** of the date that it was sent to the author requesting the change, we may have to withdraw your manuscript. We cannot publish manuscripts where authorship has not been agreed by all authors (including those who have been removed).

- Incomplete forms will be rejected.

- Please return/upload this form, fully completed, to the Journals Editorial Office. The Journal and/or Publisher will consider the information you have provided to decide whether to approve the proposed change in authorship. We may decide to contact your institution for more information or undertake a further investigation, if appropriate, before making a final decision.

SPRINGER NATURE Springer Nature is one of the world's leading global research, educational and professional publishers, created in May 2015 through the combination of Nature Publishing Group, Palgrave Macmillan, Macmillan Education and Springer Science+Business Media.

SPRINGER NATURE

Change of authorship request form - Journals (pre-acceptance)

Section 1: Please provide the current title of manuscript

Manuscript ID no.: CDD-21-1411

Title: Cullin 4b-RING ubiquitin ligase targets IRGM1 to regulate Wnt signaling and intestinal homeostasis

Section 2: Please provide the previous authorship, in the order shown on the manuscript before the changes were introduced. Please indicate the corresponding author by adding (CA) behind the name.

	First name(s)	Family name	ORCID or SCOPUS id, if available
1st author	Yujia	Fan	
2nd author	Xiaohan	Huo	
3rd author	Beibei	Guo	
4th author	Xiaohui	Zhang	
5th author	Yang	Yang	
6th author	Jiabei	Lian	
7th author	Xinyuan	Meng	
8th author	Yongxin	Zou	
9th author	Haiyang	Guo	
10th author	Haitao	Wang	

Please use an additional sheet if there are more than 10 authors.

SPRINGER NATURE Springer Nature is one of the world's leading global research, educational and professional publishers, created in May 2015 through the combination of Nature Publishing Group, Palgrave Macmillan, Macmillan Education and Springer Science+Business Media.

SPRINGER NATURE

Change of authorship request form - Journals (pre-acceptance)

Section 1: Please provide the current title of manuscript

Manuscript ID no.: CDD-21-1411

Title: Cullin 4b-RING ubiquitin ligase targets IRGM1 to regulate Wnt signaling and intestinal homeostasis

Section 2: Please provide the previous authorship, in the order shown on the manuscript before the changes were introduced. Please indicate the corresponding author by adding (CA) behind the name.

	First name(s)	Family name	ORCID or SCOPUS id, if available
11st author	Gongping	Sun	
12nd author	Hao	Dou	
13rd author	Changshun	Shao	
14th author	Yaoqin (CA)	Gong	0000-0001-6871-1560
15th author	Huili (CA)	Hu	0000-0002-1269-1887

Please use an additional sheet if there are more than 10 authors.

SPRINGER NATURE Springer Nature is one of the world's leading global research, educational and professional publishers, created in May 2015 through the combination of Nature Publishing Group, Palgrave Macmillan, Macmillan Education and Springer Science+Business Media.

SPRINGER NATURE

Change of authorship request form - Journals (pre-acceptance)

Section 3: Please provide a justification for change. Please use this section to explain your reasons for changing the authorship of your manuscript, e.g. what necessitated the change in authorship? Please refer to the (journal) policy pages for more information about authorship. Please explain why omitted authors were not originally included and/or why authors were removed on the submitted manuscript.

Yiwen Shao and Jinshen Wang were newly added, because they performed Lgr5 FISH and organoids experiments.

Section 4: Proposed new authorship. Please provide your new authorship list in the order you would like it to appear on the manuscript. Please indicate the corresponding author by adding (CA) behind the name. <u>If the Corresponding Author has changed, please indicate the reason under section 3</u>.

	First name(s)	Family name (this name will appear in full on the final publication and will be searchable in various abstract and indexing databases)	Affiliated institute	E-mail address
1st author	Yujia	Fan	Shandong University Cheeloo Medical College, School of Basic Medical Sciences	fanyujia_2008@163.com
2nd author	Xiaohan	Huo	Shandong University Cheeloo Medical College, School of Basic Medical Sciences	nt9607@163.com
3rd author	Beibei	Guo	Shandong University Cheeloo Medical College, School of Basic Medical Sciences	g93565375bb@163.com
4th author	Xiaohui	Zhang	Shandong University Cheeloo Medical College, School of Basic Medical Sciences	1657385984@qq.com
5th author	Yang	Yang	Shandong University Cheeloo Medical College, School of Basic Medical Sciences	xyyf1985@163.com
6th author	Jiabei	Lian	Shandong University Cheeloo Medical College, School of Basic Medical Sciences	lianjiabei1995@163.com
7th author	Xinyuan	Meng	Shandong University Cheeloo Medical College, School of Basic Medical Sciences	partyona@163.com
8th author	Yiwen	Shao	Shandong University Cheeloo Medical College, School of Basic Medical Sciences	202015435@mail.sdu.edu.cn
9th author	Yongxin	Zou	Shandong University Cheeloo Medical College, School of Basic Medical Sciences	zouyongxin@sdu.edu.cn
10th author	Haiyang	Guo	Department of Clinical Laboratory, the Second Hospital of Shandong University	allen100006@163.com

Please use an additional sheet if there are more than 10 authors.

SPRINGER NATURE Springer Nature is one of the world's leading global research, educational and professional publishers, created in May 2015 through the combination of Nature Publishing Group, Palgrave Macmillan, Macmillan Education and Springer Science+Business Media.

SPRINGER NATURE

Change of authorship request form - Journals (pre-acceptance)

Section 3: Please provide a justification for change. Please use this section to explain your reasons for changing the authorship of your manuscript, e.g. what necessitated the change in authorship? Please refer to the (journal) policy pages for more information about authorship. Please explain why omitted authors were not originally included and/or why authors were removed on the submitted manuscript.

Section 4: Proposed new authorship. Please provide your new authorship list in the order you would like it to appear on the manuscript. Please indicate the corresponding author by adding (CA) behind the name. <u>If the Corresponding Author has changed, please indicate the reason under section 3</u>.

	First name(s)	Family name (this name will appear in full on the final publication and will be searchable in various abstract and indexing databases)	Affiliated institute	E-mail address
11st author	Haitao	Wang	Department of Pathology, the Second Hospital of Shandong University	what200488@126.com
12nd author	Gongping	Sun	Shandong University Cheeloo Medical College, School of Basic Medical Sciences	sgp@sdu.edu.cn
13rd author	Hao	Dou	Shandong University Cheeloo Medical College, School of Basic Medical Sciences	douhao@sdu.edu.cn
14th author	Jinshen	Wang	Department of Gastrointestinal Surgery Shandong Provincial Hospital Affiliated to Shandong First Medical University	doctorwjs@163.com
15th author	Changshun	Shao	The State Key Laboratory of Radiation Medicine and Protection, Institutes for Translational Medicine, Soochow University	shaoc@suda.edu.cn
16th author	Yaoqin (CA)	Gong	Shandong University Cheeloo Medical College, School of Basic Medical Sciences	yxg8@sdu.edu.cn
17th author	Huili (CA)	Hu	Shandong University Cheeloo Medical College, School of Basic Medical Sciences	huhuili@sdu.edu.cn

Please use an additional sheet if there are more than 10 authors.

SPRINGER NATURE Springer Nature is one of the world's leading global research, educational and professional publishers, created in May 2015 through the combination of Nature Publishing Group, Palgrave Macmillan, Macmillan Education and Springer Science+Business Media.

SPRINGER NATURE

Change of authorship request form - Journals (pre-acceptance)

Section 5: Author contribution, Acknowledgement and Disclosures. Please use this section to provide a new disclosure statement and, if appropriate, acknowledge any contributors who have been removed as authors and ensure you state what contribution any new authors made (if applicable per the journal or book (series) policy). Please ensure these are updated in your manuscript - after approval of the change(s) - as our production department will not transfer the information in this form to your manuscript.

New acknowledgements:

Not applicable

New Disclosures (financial and non-financial interests, funding):

Conflict of Interest

The authors declare that they have no conflict of interest.

Funding

The Shandong Provincial Natural Science Foundation ZR2020MH221 (J.S.)

New Author Contributions statement (if applicable per the journal policy):

J.W. and Y.S. helped to set up organoids and experiments.

State 'Not applicable' if there are no new authors.

SPRINGER NATURE Springer Nature is one of the world's leading global research, educational and professional publishers, created in May 2015 through the combination of Nature Publishing Group, Palgrave Macmillan, Macmillan Education and Springer Science+Business Media.

SPRINGER NATURE

Change of authorship request form - Journals (pre-acceptance)

Section 6: Declaration of agreement. *All* authors, unchanged, new and removed *must* sign this declaration.
(NB: Please print the form, (docu)-sign and return/upload a scanned copy. Please note that signatures that have been inserted as an image file are acceptable as long as it is handwritten. Typed names in the signature box are unacceptable.) *** Please delete as appropriate. Delete all of the bold if you were on the original authorship list and are remaining as an author.**

	First name	Family name		Signature	Date
1st author	Yujia	Fan	I agree to the proposed new authorship shown in section 4 **/and the addition/removal*of my name to the authorship list** /and the proposed change in corresponding author	Yujia Fan	2022.2.5
2nd author	Xiaohan	Huo	I agree to the proposed new authorship shown in section 4 **/and the addition/removal*of my name to the authorship list** /and the proposed change in corresponding author	Xiaohan Huo	22.2.5
3rd author	Beibei	Guo	I agree to the proposed new authorship shown in section 4 **/and the addition/removal*of my name to the authorship list** /and the proposed change in corresponding author	Beibei Guo	2022.2.5
4th authors	Xiaohui	Zhang	I agree to the proposed new authorship shown in section 4 **/and the addition/removal*of my name to the authorship list** /and the proposed change in corresponding author	Xiaohui Zhang	2022.2.5
5th author	Yang	Yang	I agree to the proposed new authorship shown in section 4 **/and the addition/removal*of my name to the authorship list** /and the proposed change in corresponding author	Yang YANG	2022.2.5
6th author	Jiabei	Lian	I agree to the proposed new authorship shown in section 4 **/and the addition/removal*of my name to the authorship list** /and the proposed change in corresponding author	Jiabei Lian	2022.2.5
7th author	Xinyuan	Meng	I agree to the proposed new authorship shown in section 4 **/and the addition/removal*of my name to the authorship list** /and the proposed change in corresponding author	Xinyuan Meng	2022.2.5

SPRINGER NATURE Springer Nature is one of the world's leading global research, educational and professional publishers, created in May 2015 through the combination of Nature Publishing Group, Palgrave Macmillan, Macmillan Education and Springer Science+Business Media.

SPRINGER NATURE

Change of authorship request form - Journals (pre-acceptance)

Section 6: Declaration of agreement. *All* authors, unchanged, new and removed *must* sign this declaration.
(NB: Please print the form, (docu)-sign and return/upload a scanned copy. Please note that signatures that have been inserted as an image file are acceptable as long as it is handwritten. Typed names in the signature box are unacceptable.) *** Please delete as appropriate. Delete all of the bold if you were on the original authorship list and are remaining as an author.**

	First name	Family name		Signature	Date
8th author	Yiwen	Shao	I agree to the proposed new authorship shown in section 4 **/and the addition/removal*of my name to the authorship list** /and the proposed change in corresponding author	Yiwen Shao	2022. 2. 5
9th author	Yongxin	Zou	I agree to the proposed new authorship shown in section 4 **/and the addition/removal*of my name to the authorship list** /and the proposed change in corresponding author	Zou Yongxin	2022.2.5
10th author	Haiyang	Guo	I agree to the proposed new authorship shown in section 4 **/and the addition/removal*of my name to the authorship list** /and the proposed change in corresponding author	Haiyang Guo	2022. 2. 4
11st author	Haitao	Wang	I agree to the proposed new authorship shown in section 4 **/and the addition/removal*of my name to the authorship list** /and the proposed change in corresponding author	Haitao Wang	2022.2.5
12nd author	Gongping	Sun	I agree to the proposed new authorship shown in section 4 **/and the addition/removal*of my name to the authorship list** /and the proposed change in corresponding author	[illegible]	2022.2.4
13rd author	Hao	Dou	I agree to the proposed new authorship shown in section 4 **/and the addition/removal*of my name to the authorship list** /and the proposed change in corresponding author	Hao Dou	2022.2.5
14th authors	Jinshen	Wang	I agree to the proposed new authorship shown in section 4 **/and the addition/removal*of my name to the authorship list** /and the proposed change in corresponding author	Jinshen Wang	2022 - 2 - 5

SPRINGER NATURE Springer Nature is one of the world's leading global research, educational and professional publishers, created in May 2015 through the combination of Nature Publishing Group, Palgrave Macmillan, Macmillan Education and Springer Science+Business Media.

SPRINGER NATURE

Change of authorship request form - Journals (pre-acceptance)

	First name	Family name		Signature	Date
15th author	Changshun	Shao	I agree to the proposed new authorship shown in section 4 /**and the addition/removal*of my name to the authorship list** /and the proposed change in corresponding author	Chang Shao	2022.2.5
16th author	Yaoqin	Gong	I agree to the proposed new authorship shown in section 4 /**and the addition/removal*of my name to the authorship list** /and the proposed change in corresponding author	Yaoqin Gong	2022.2.4
17th author	Huili	Hu	I agree to the proposed new authorship shown in section 4 /**and the addition/removal*of my name to the authorship list** /and the proposed change in corresponding author	Huili Hu	2022.2.5

Please use an additional sheet if there are more than 10 authors.

In case of author collaborations with formal agreement:

	Name of consortium/consortia	First name	Family name		Signature	Date
Representative/legal guarantor				I agree to the proposed new authorship shown in section 4 /**and the addition/removal*of my name to the authorship list** /and the proposed change in corresponding author		

Both added/removed authors should complete the information in the first table under Section 6.

---- End of form ----

SPRINGER NATURE Springer Nature is one of the world's leading global research, educational and professional publishers, created in May 2015 through the combination of Nature Publishing Group, Palgrave Macmillan, Macmillan Education and Springer Science+Business Media.
