# Supplementary material for: Cullin 4b-RING ubiquitin ligase targets IRGM1 to regulate Wnt signaling and intestinal homeostasis
Source: Cell Death Differ. 2022 Feb 23;29(9):1673–88. doi: 10.1038/s41418-022-00954-9 (PMC9433385; doi:10.1038/s41418-022-00954-9)

Figure 1b

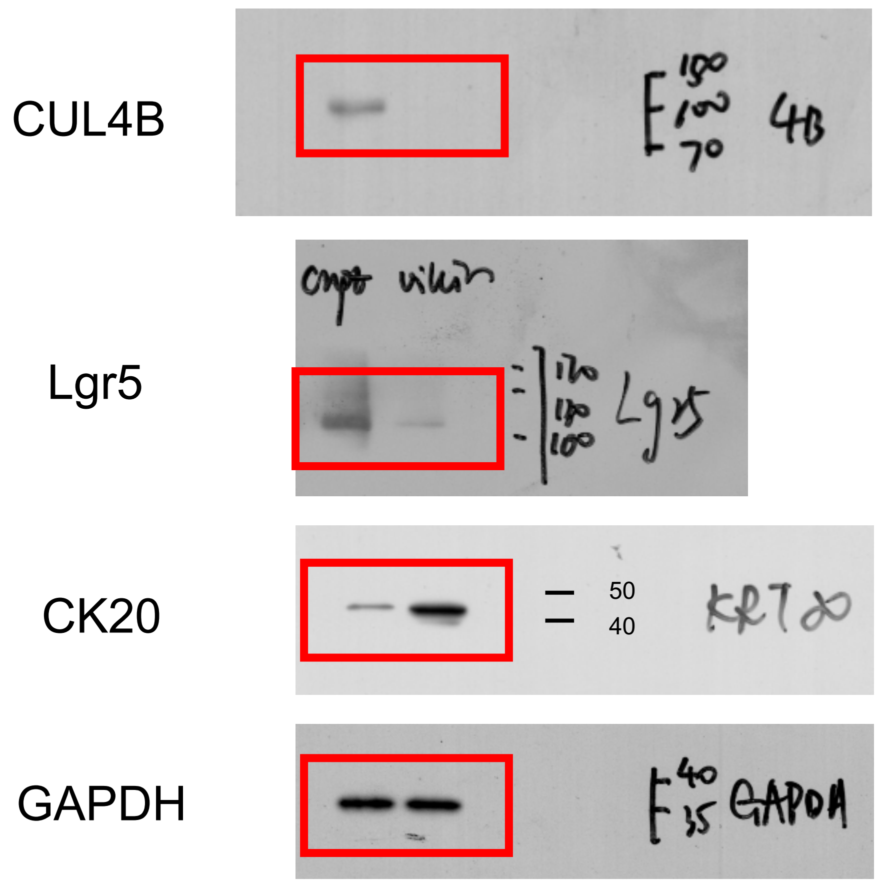

Figure 1c

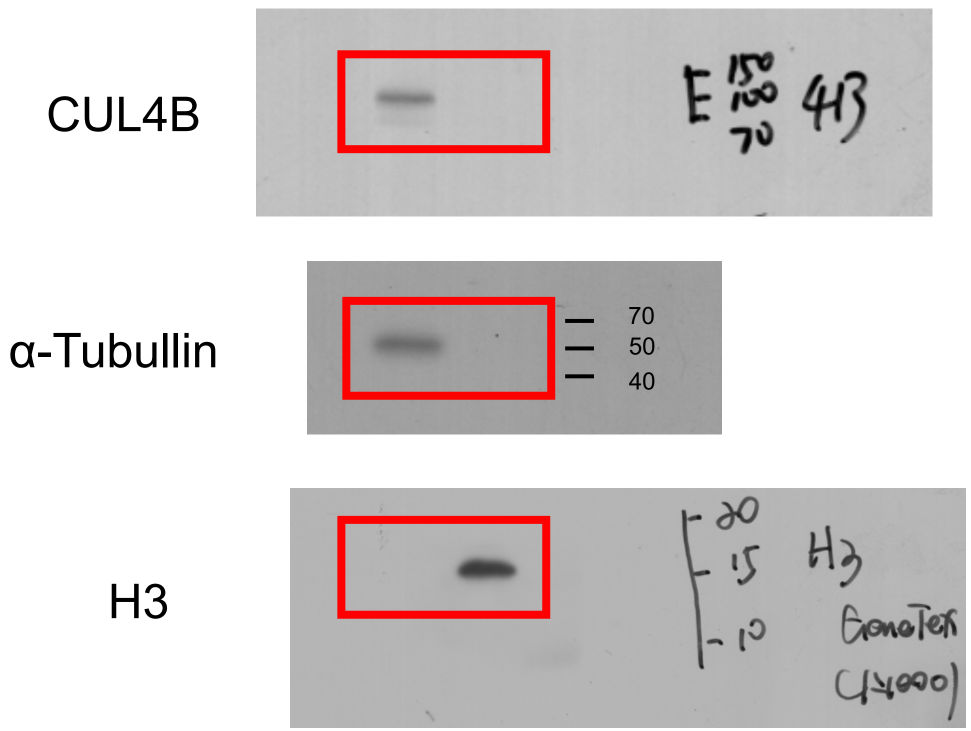

Figure 4b

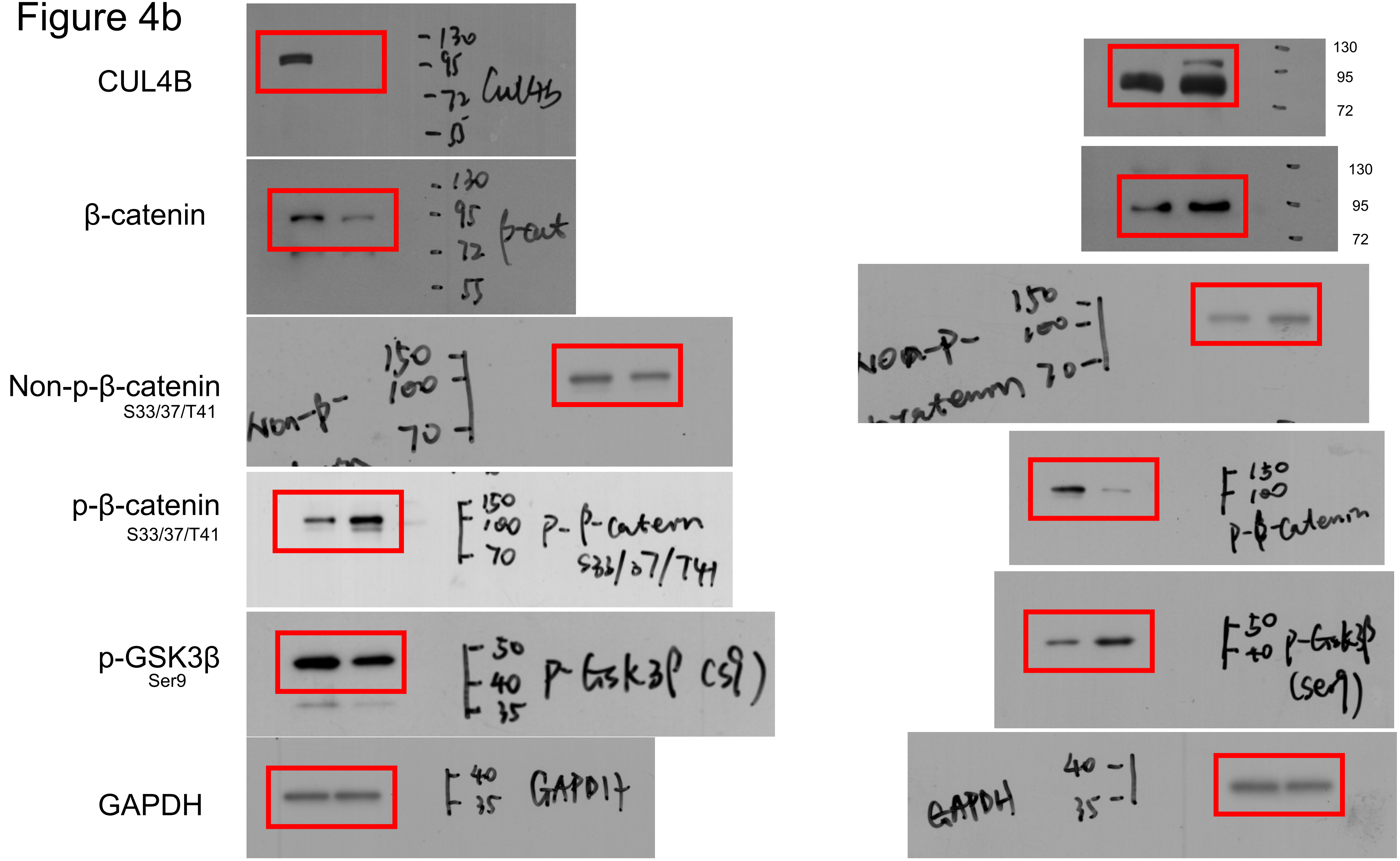

CUL4B
β-catenin
Non-p-β-catenin
S33/37/T41
p-β-catenin
S33/37/T41
p-GSK3β
Ser9
GAPDH
130
95
72
130
95
72

Figure 4d

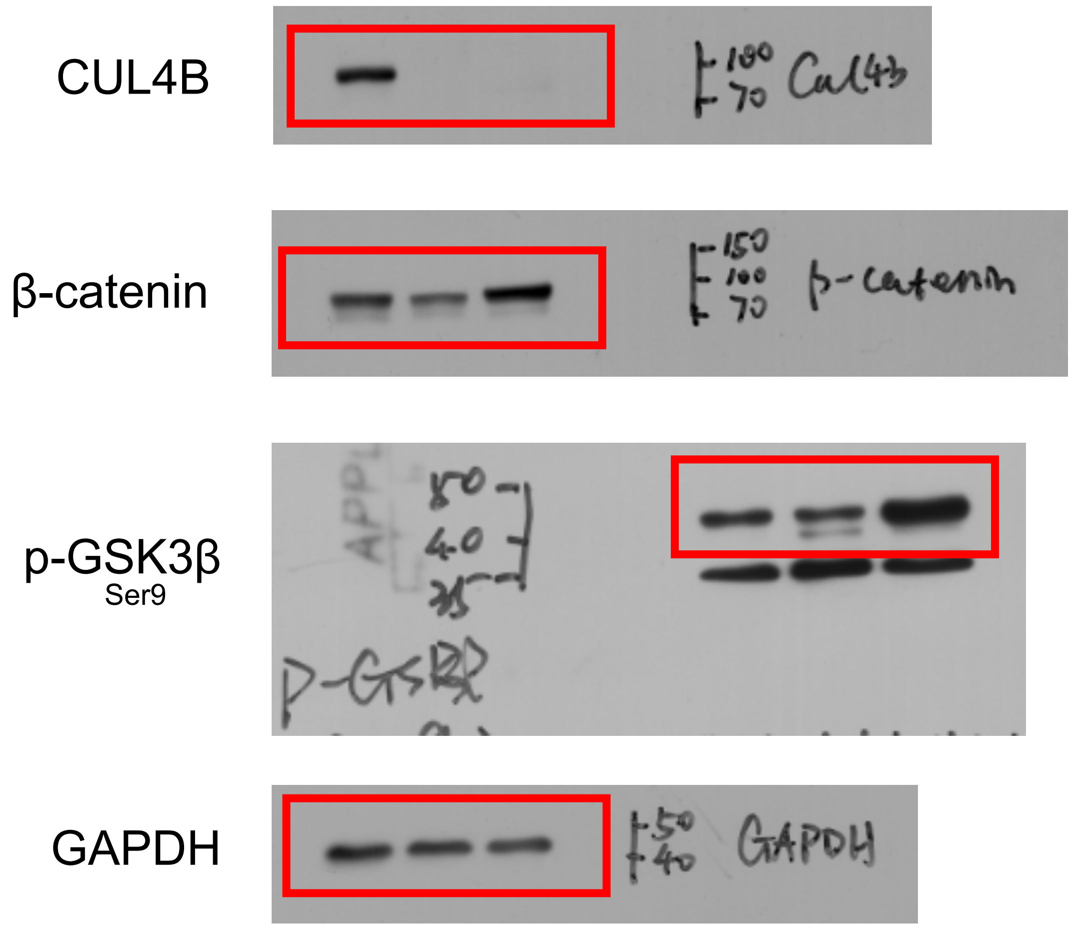

Figure 5h

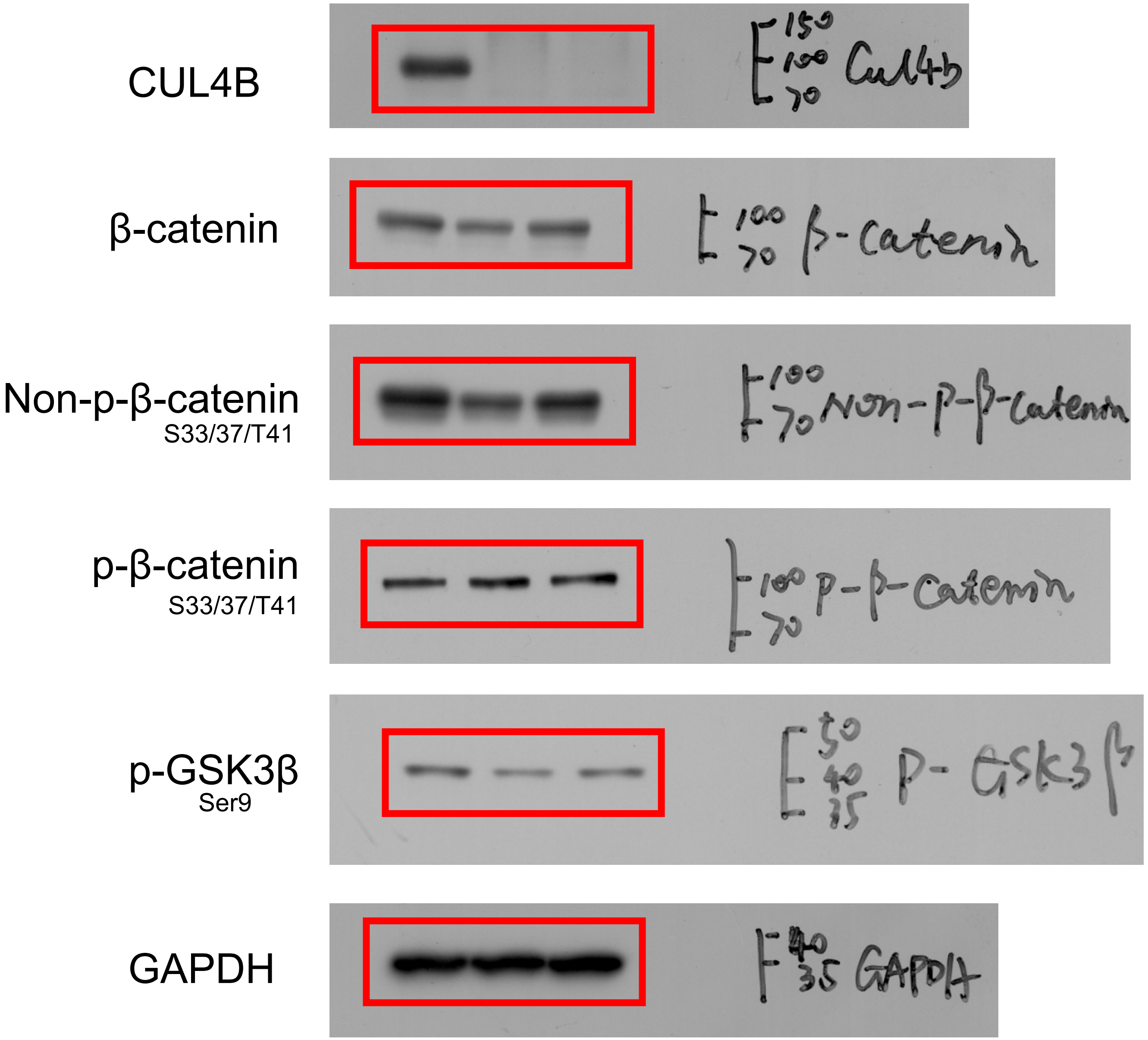

CUL4B
β-catenin
Non-p-β-catenin
S33/37/T41
p-β-catenin
S33/37/T41
p-GSK3β
Ser9
GAPDH

Figure 6d

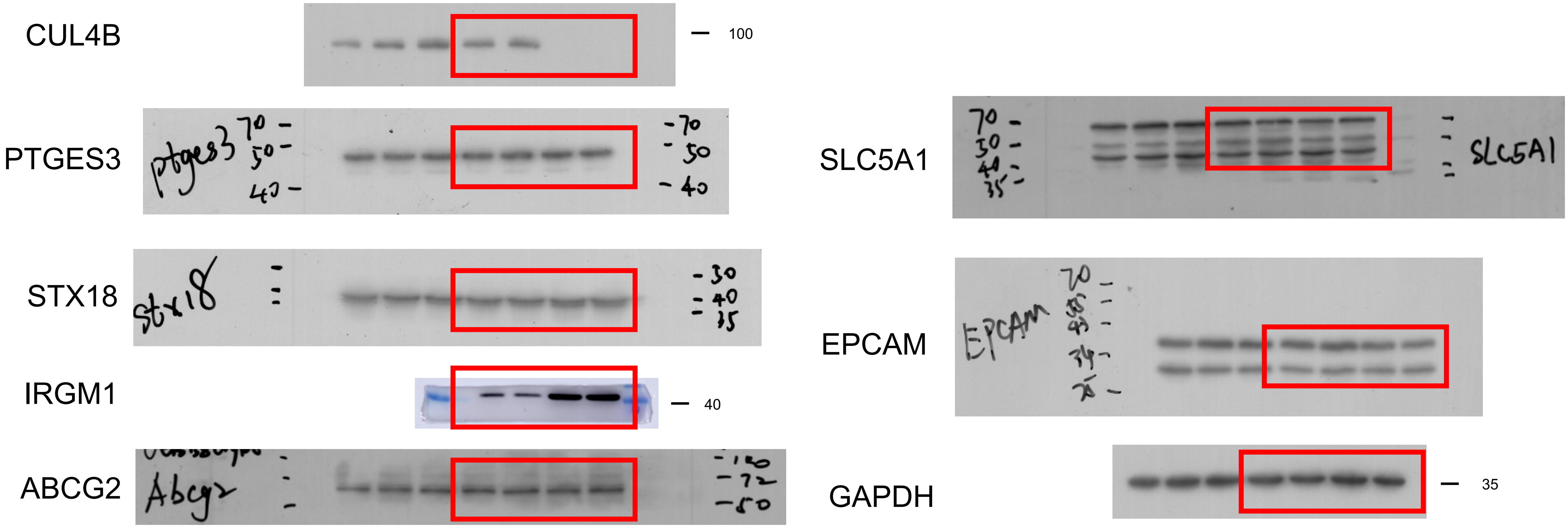

Figure 6e

CUL4B

IRGM1

GAPDH

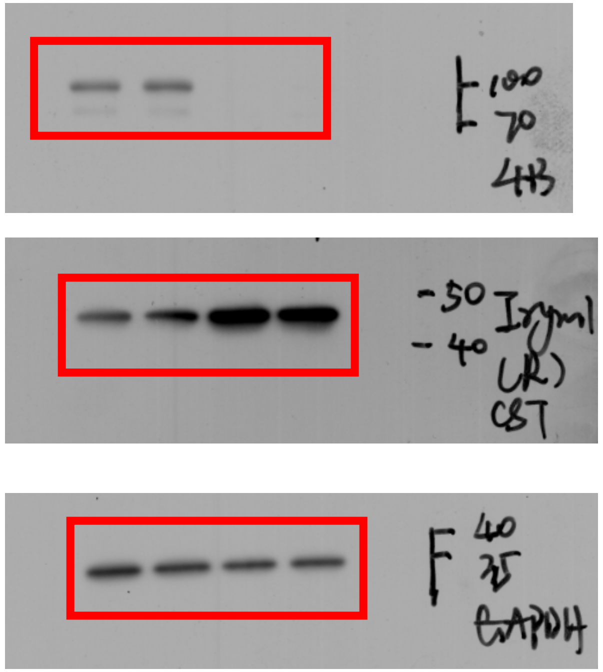

Figure 6f

CUL4B

IRGM1

GAPDH

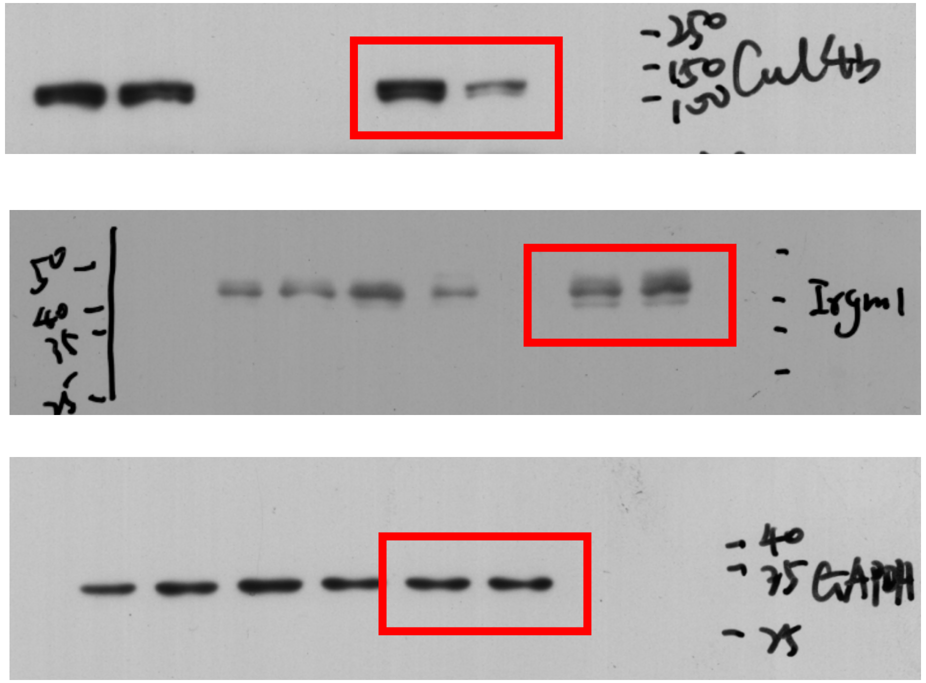

Figure 6g

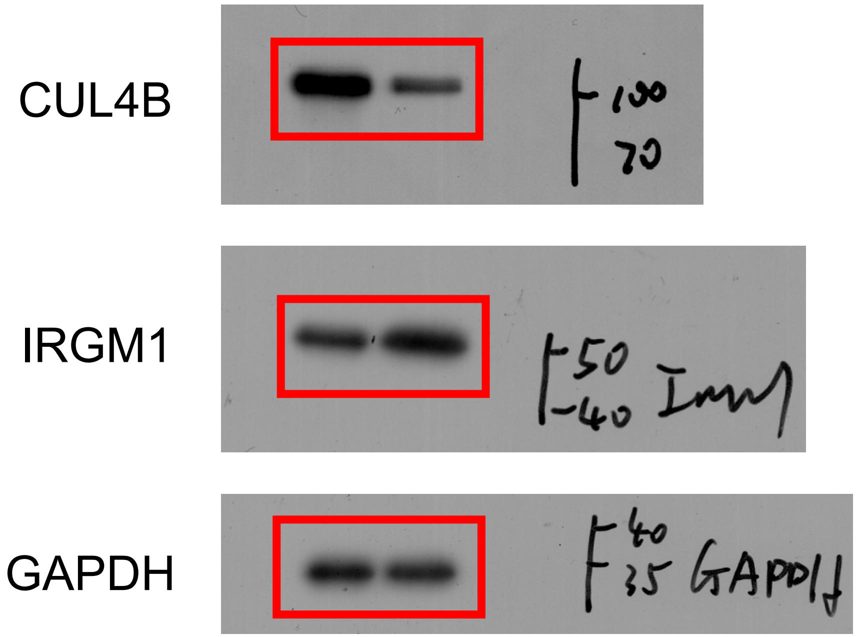

Figure 6h

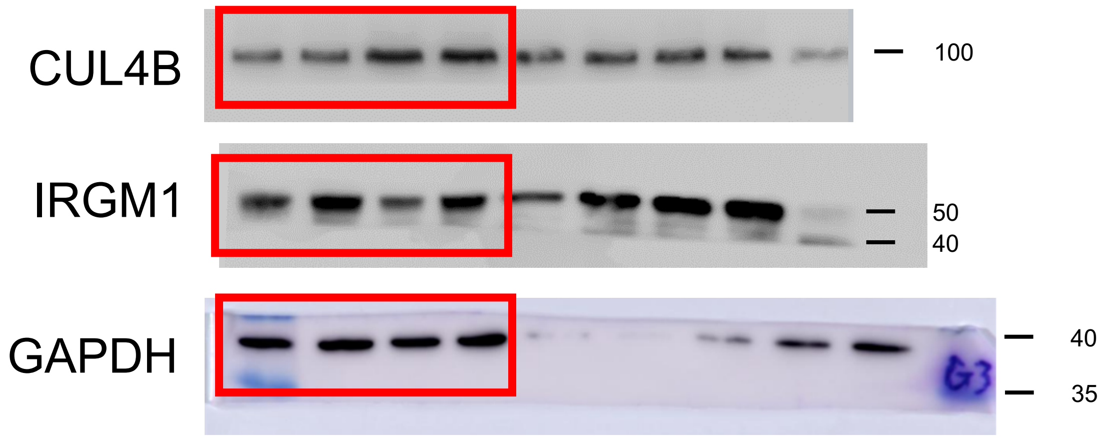

Figure 6i

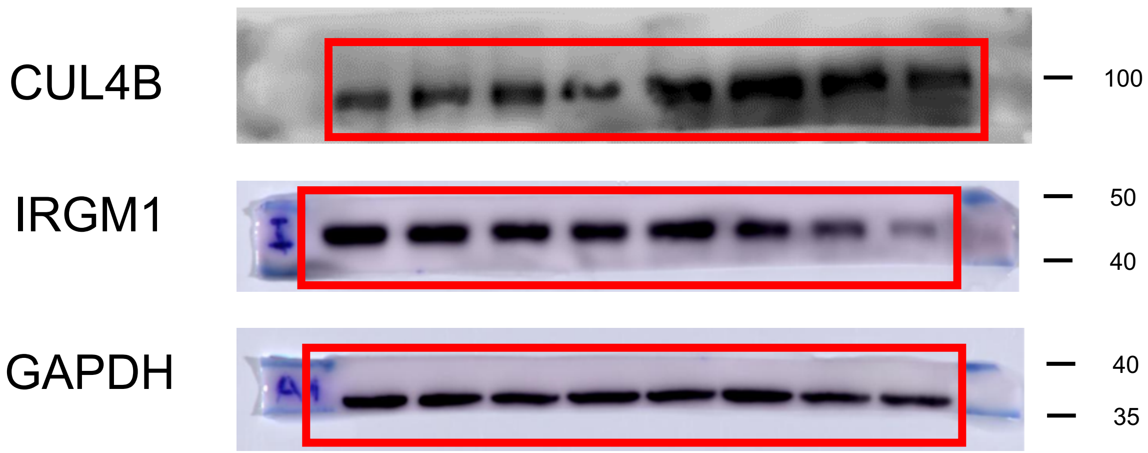

Figure 7a

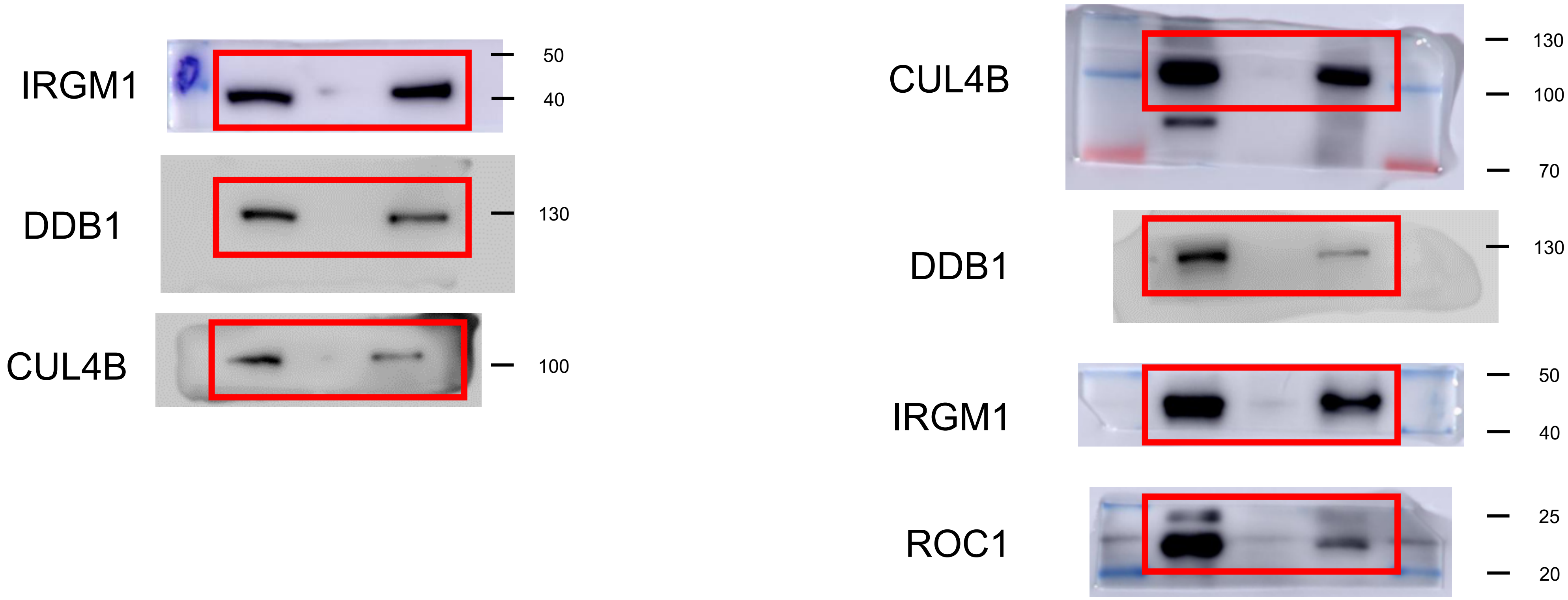
IRGM1
50
40
DDB1
130
CUL4B
100
CUL4B
130
100
70
DDB1
130
IRGM1
50
40
ROC1
25
20

Figure 7b

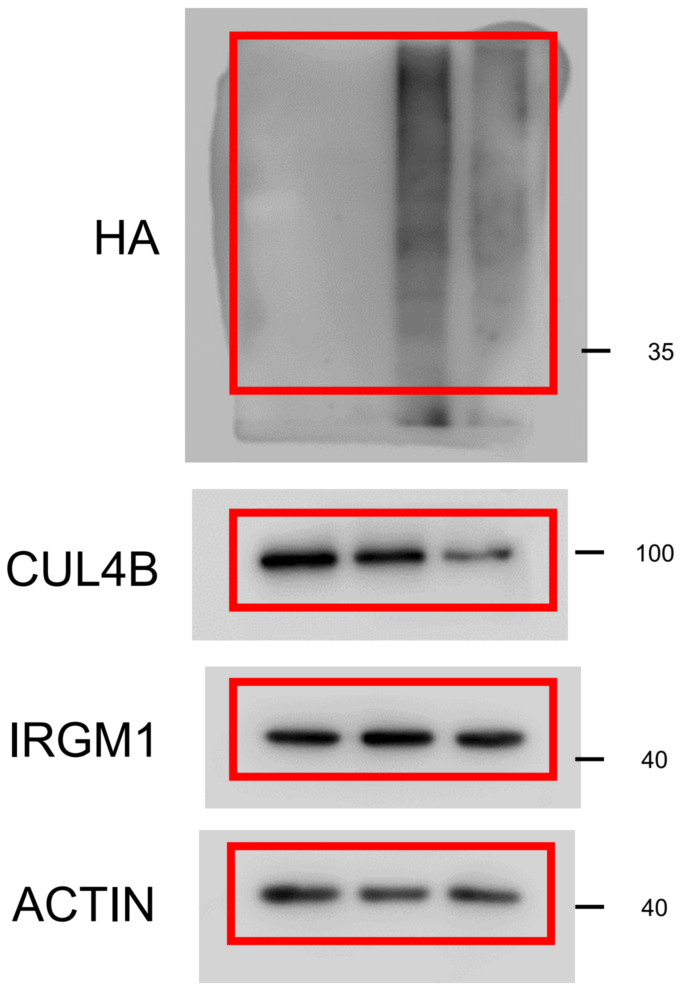

Figure 7c

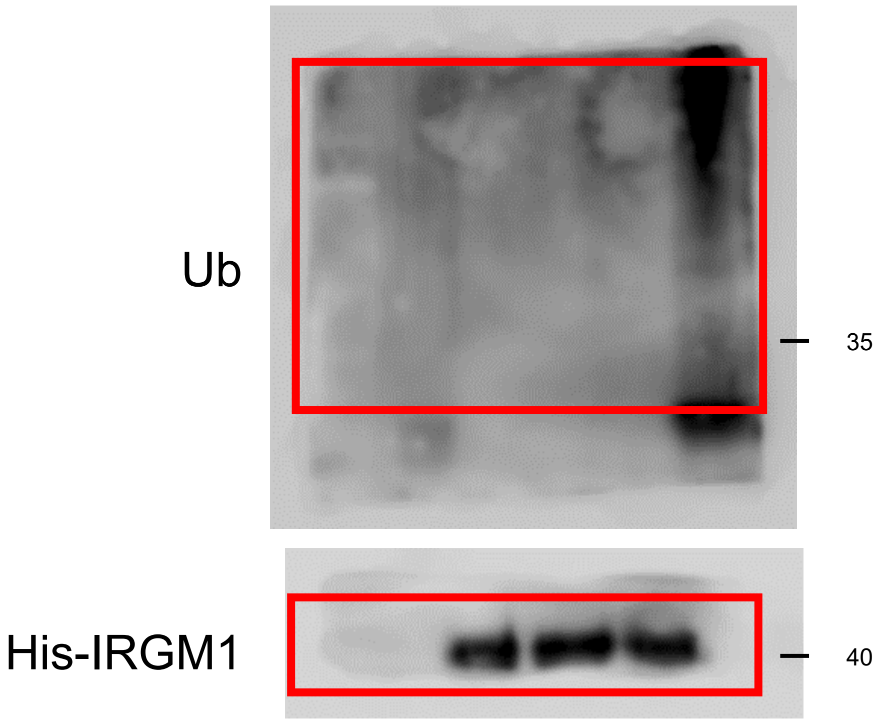

Figure 7d

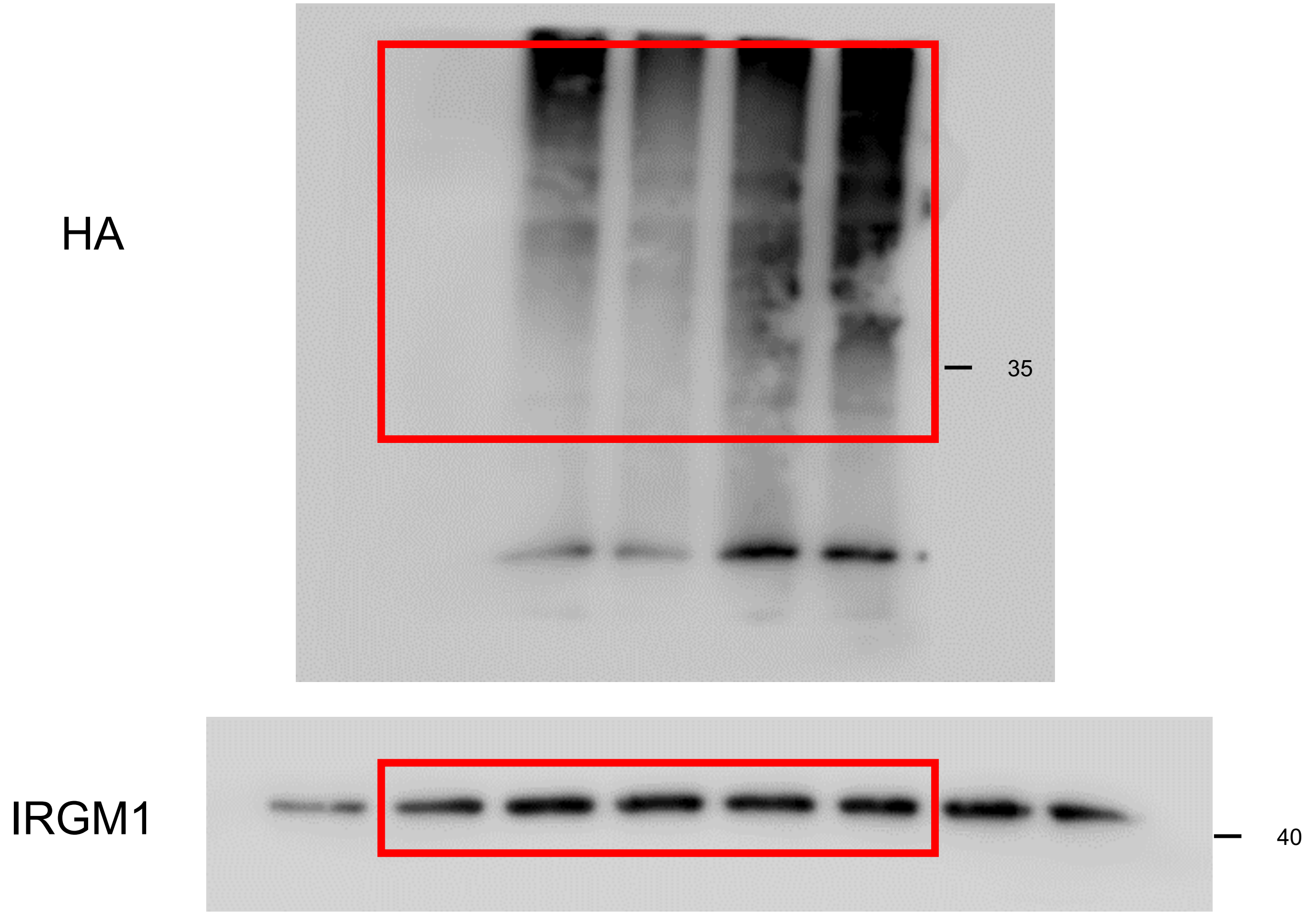

Figure 7e

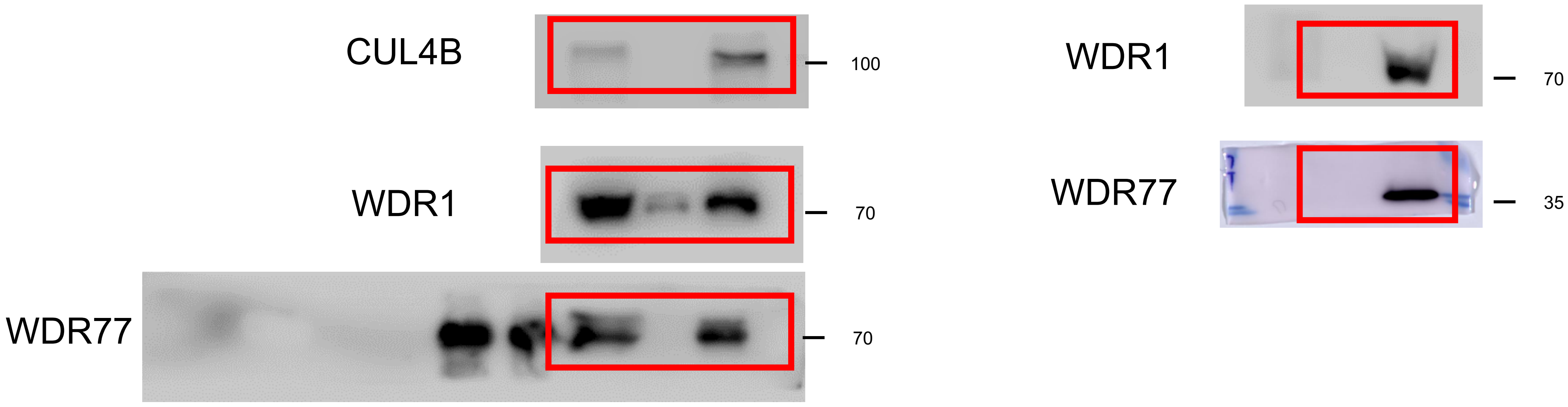

Figure 7f

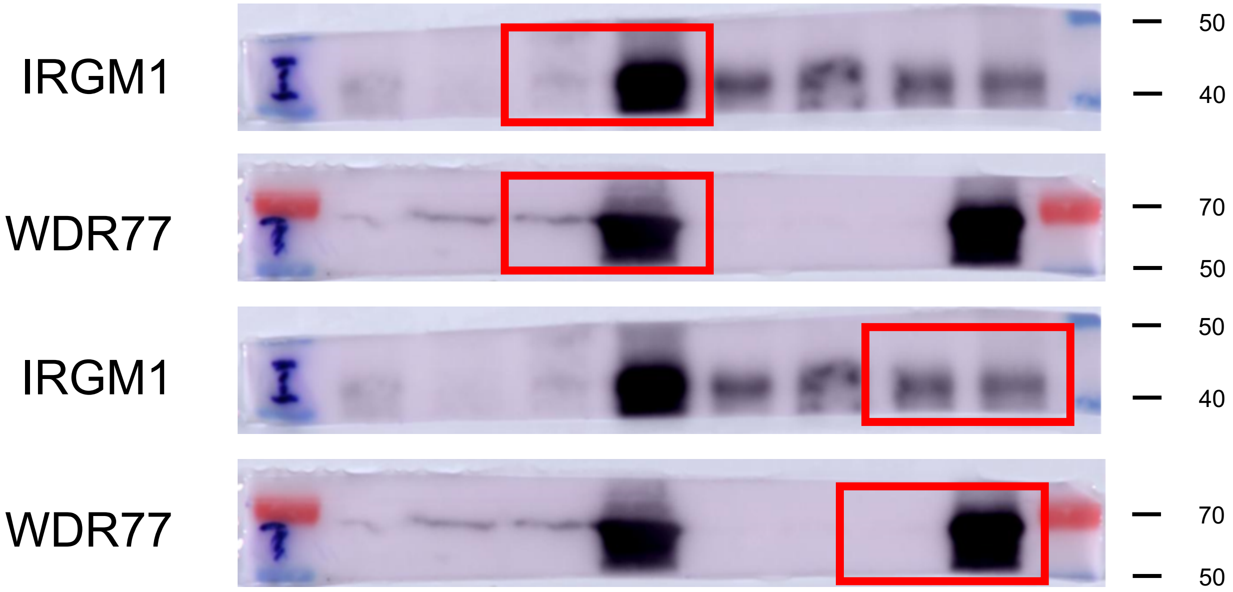

Figure 7g

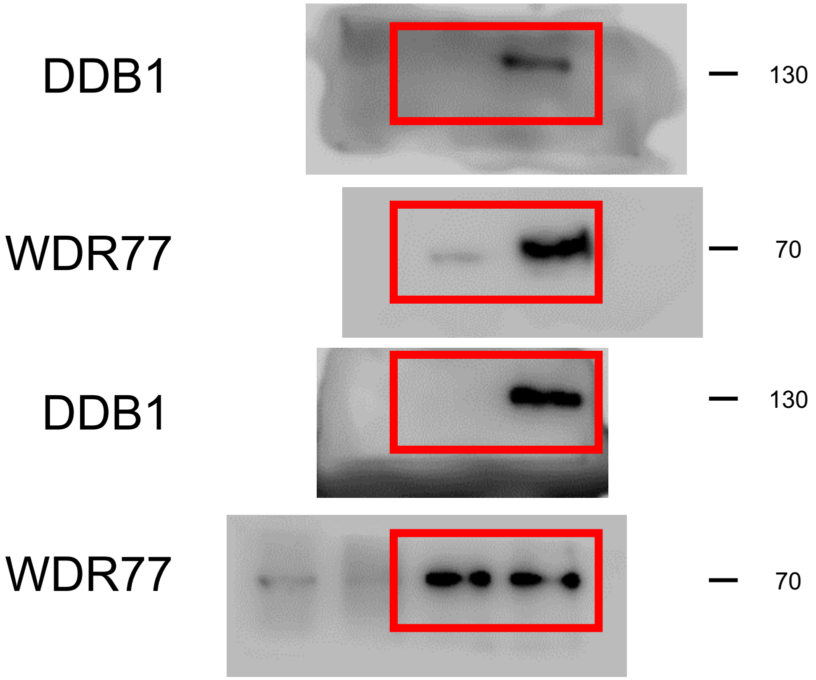

Figure 7h

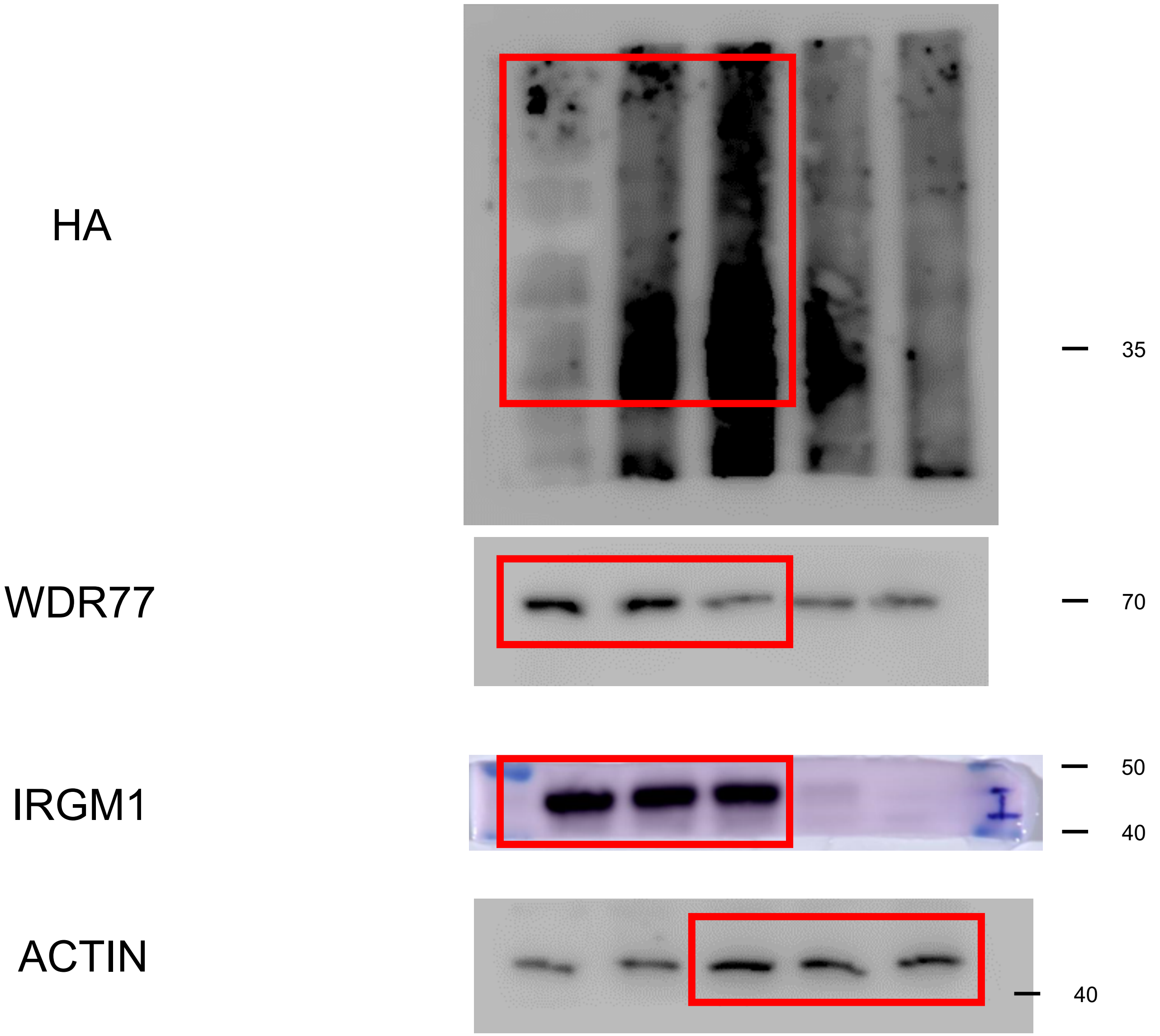

Figure 8j

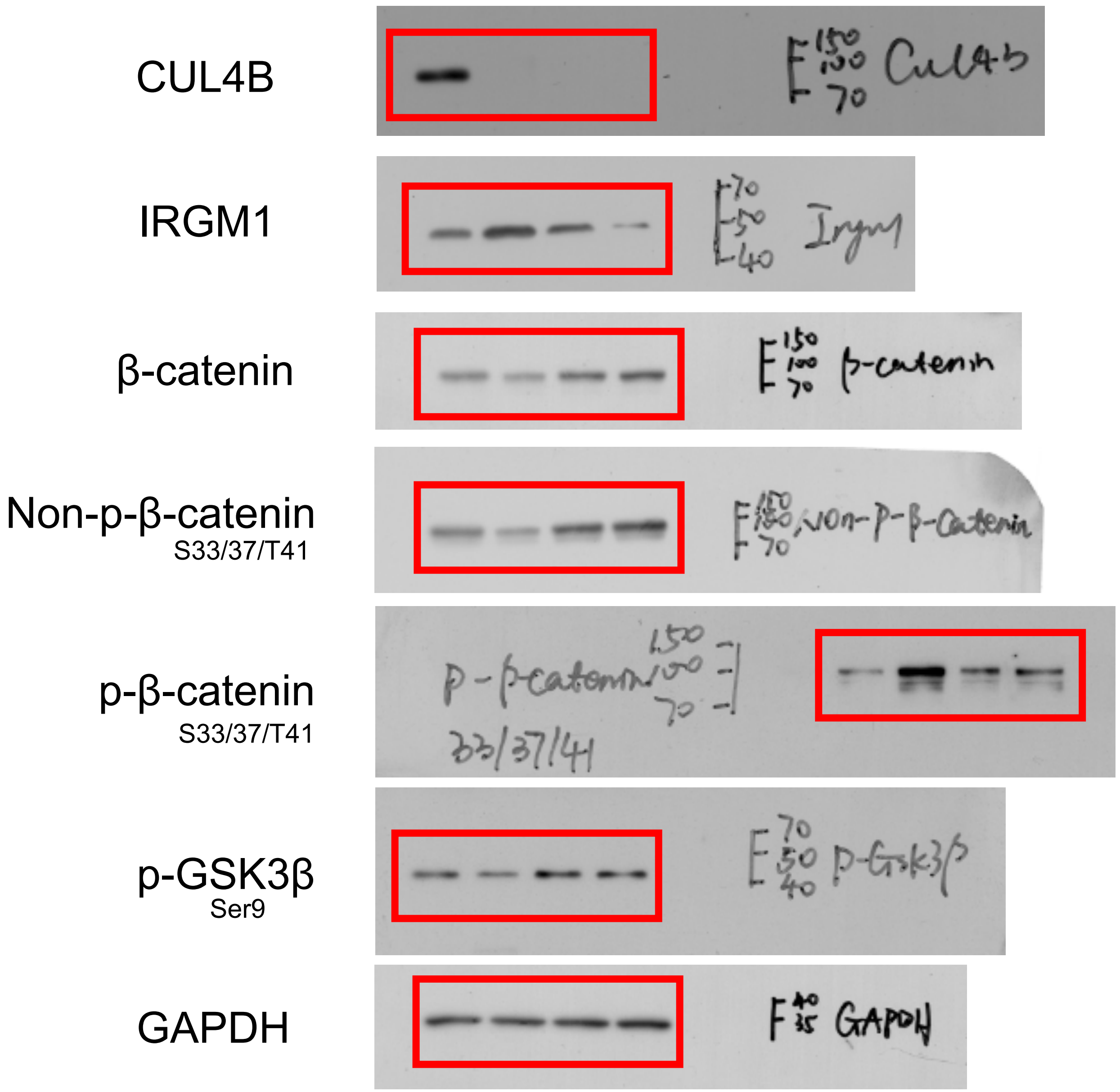
CUL4B
IRGM1
β-catenin
Non-p-β-catenin
S33/37/T41
p-β-catenin
S33/37/T41
p-GSK3β
Ser9
GAPDH
150
100
70
Cul4b
70
50
40
Irgm1
150
100
70
β-catenin
150
100
70
non-P-β-Catenin
p-β-catenin
33/37/41
150
100
70
70
50
40
p-Gsk3β
40
35
GAPDH

Figure S1c

CUL4B

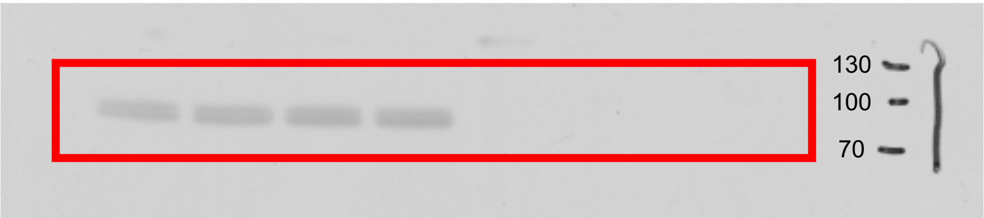

GAPDH

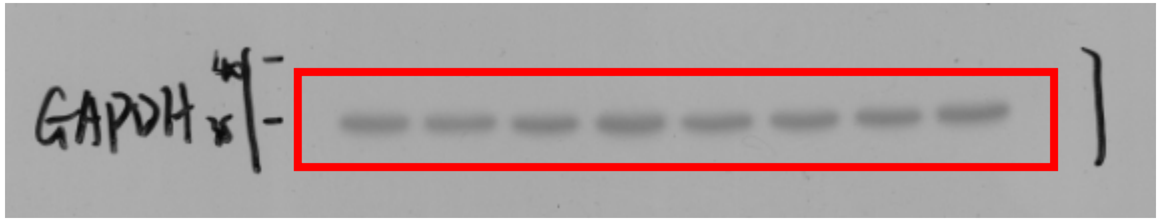

Figure S2b

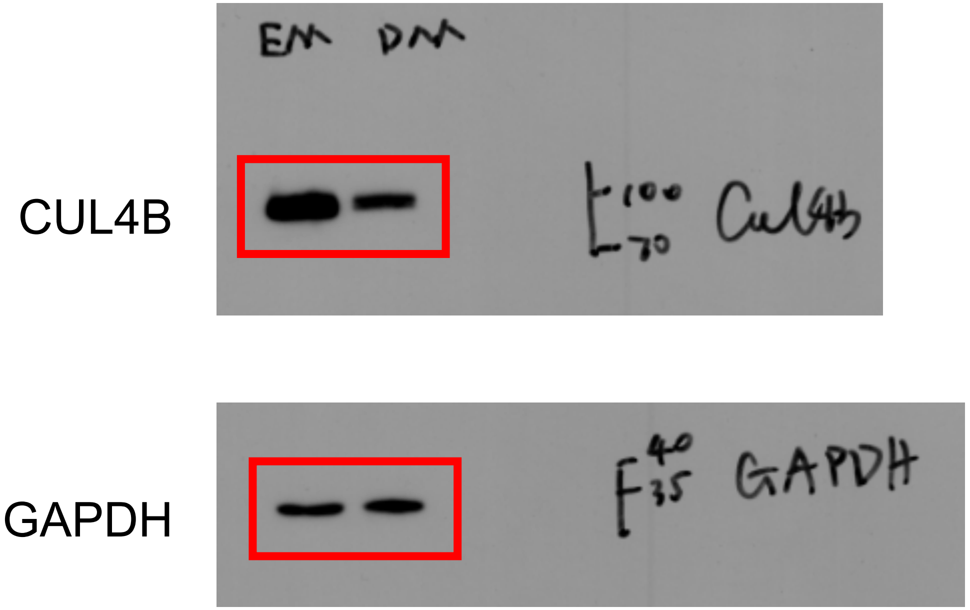

Figure S6b

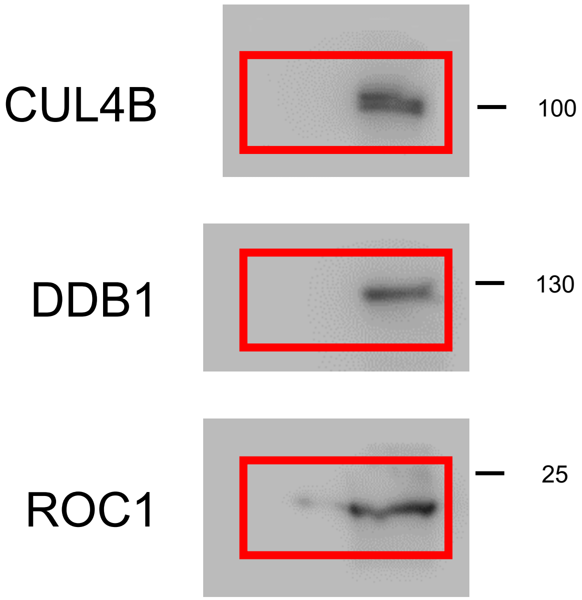

Supplement: Supplementary file 3 — Supplementary materials for WB [file 41418_2022_954_MOESM3_ESM.pdf]
