# Supplementary material for: Cullin 4b-RING ubiquitin ligase targets IRGM1 to regulate Wnt signaling and intestinal homeostasis
Source: Cell Death Differ. 2022 Feb 23;29(9):1673–88. doi: 10.1038/s41418-022-00954-9 (PMC9433385; doi:10.1038/s41418-022-00954-9)
Supplement: Supplementary file 4 — Table S1 The list of primers used for qRT-PCR. [file 41418_2022_954_MOESM4_ESM.docx]

Supplementary Table 1. qRT-PCR primers

| **Mouse gene** | **Forward primer 5’-3’** | **Reverse primer 5’-3’** |
| --- | --- | --- |
| Gapdh | AGGTCGGTGTGAACGGATTTG | GGGGTCGTTGATGGCAACA |
| Cul4b | TATTAGTTGGCAAGAGTGCAT | CCAGTAACCCATTGTCAGGAT |
| Lgr5 | CGGGACCAGATGCGATAC | GAAGCAGAGGCGATGTAGGA |
| Ascl2 | AAGCACACCTTGACTGGTACG | AAGTGGACGTTTGCACCTTCA |
| Lrg1 | AGGAAGCCTCCAGGATCTCA | GGACAGTGTCGGCAGGGA |
| Rspo1 | ATCGGAAGAGCGGACACG | TGCTCCTTGCTGTTCTTCCTG |
| Igfbp5 | AGATGAGACAGGAATCCGAACA | CCACGGGAGGGCTTACACT |
| Alpi | ATGATGCCAACCGAAACCC | CAAACTCAGTCAGTGCCAGGTAA |
| Vil1 | AGACCCACCCTGGAAACCT | CGCATAACCTCGTCAGCAAT |
| Krt20 | AGCACCATCCGAGACTATCCC | CGGCTTCCACAGCTATACGC |
| Bmp1 | TTGTACGCGAGAACATACAGC | CTGAGTCGGGTCCTTTGGC |
| Apoa4 | CTGGTGCCCTTTGTCGTAGA | CCGTCTGGGTTACTTTGTTGG |
| Lct | ACAACAGCACATACCGTAACGA | TGAATCCGTGTCTTCATCACC |
| Igf2 | GCTTGTTGACACGCTTCAGTT | AAAGCAGCACTCTTCCACGAT |
| Axin2 | ACCGTGGTTGGCTTGTCC | TCAGTGCGTCGCTGGATAA |
| c-Myc | ACAATCTGCGAGCCAGGACA | GGGCATCGTCGTGGCTG |
| Cyclin D1 | CGCCCTCCGTATCTTACTTCA | CTTCGCACTTCTGCTCCTCAC |
| Ephb2 | GCGGCTACGACGAGAACAT | GGCTAAGTCAAAATCAGCCTCA |
| Ets2 | CCTGTCGCCAACAGTTTTCG | TGGAGTGTCTGATCTTCACTGA |
| Prom1 | GTTGAGACTGTGCCCATGAAA | GACGGGCTTGTCATAACAGGA |
| Sox9 | CAAGCGGAGGCCGAAGA | CAGCTTGCACGTCGGTTT |
| Il10 | CCTGCCTAACATGCTTCGAG | GAGTTCACATGCGCCTTGAT |
| Il-1β | GCTGAAGGAGTTGCCAGAAA | GTGCAAGTGACTCAGGGTGA |
| Tnfα | CTGAAAGCATGATCCGGGAC | TTAGAGAGAGGTCCCTGGGG |
| Il6 | GTTGTTTGTGAGTGGGGTCC | CGACGCACATGGACACTATG |
| Akp3 | CCCCTCAATGCTCTGGAC | CGTGGGTCTCCGACTTCA |
| ChgA | ATCCTCTCTATCCTGCGACAC | GGGCTCTGGTTCTCAAACACT |
| Klf4 | GGAGCCCAAGCCAAAGAG | GTCCCAGTCACAGTGGTAAGGT |
| Lyz1 | GGAATGGATGGCTACCGTGG | CATGCCACCCATGCTCGAAT |
| Defa1 | AAGAGACTAAAACTGAGGAGCAGC | CGACAGCAGAGCGTGTA |
| Defa4 | CCAGGGGAAGATGACCAGGCTG | TGCAGCGACGATTTCTACAAAGGC |
| Defa5 | AGGCTGATCCTATCCACAAAACAG | TGAAGAGCAGACCCTTCGGC |
| Camp | GCTGTGGCGGTCACTATCAC | TGTCTAGGGACTGCTGGTTGA |
