# Supplementary material for: Cullin 4b-RING ubiquitin ligase targets IRGM1 to regulate Wnt signaling and intestinal homeostasis
Source: Cell Death Differ. 2022 Feb 23;29(9):1673–88. doi: 10.1038/s41418-022-00954-9 (PMC9433385; doi:10.1038/s41418-022-00954-9)
Supplement: Supplementary file 8 — Table S5 The reagents and resource we used in the manuscripts. [file 41418_2022_954_MOESM8_ESM.docx]

| **REAGENT or RESOURCE** | **SOURCE** | **IDENTIFIER** |
| --- | --- | --- |
| **Antibodies** | | |
| anti-CUL4B | Sigma-Aldrich | C9995 |
| anti-Lgr5 | BBI Life Sciences | D220920 |
| anti-Histone | GeneTex | GTX122148 |
| Rat anti-CD24 | Abcam | ab64064 |
| Cytokeratin 20 | GeneTex | GTX110600 |
| Histone H3 | GeneTex | GTX122148 |
| anti-Gapdh | Cell Signaling Technology | 5174S |
| anti-GFP | ROCKLAND | 600-101-215S |
| anti-PCNA | GeneTex | GTX100539 |
| Anti-BrdU | Abcam | ab6326 |
| Rabbit anti-Ki67 | Abcam | ab15580 |
| anti-Olfm4 | Cell Signaling Technology | 14369 |
| anti-β-catenin | Santa Cruz | SC7963 |
| anti-Non-p-β-catenin | Cell Signaling Technology | 8814S |
| Phospho-GSK-3β (Ser9) (5B3) Rabbit | Cell Signaling Technology | 9323P |
| Phospho-β-Catenin (Ser33/37/Thr41) | Cell Signaling Technology | 9561S |
| anti-Irgm1 | GeneTex | GTX53610 |
| anti-IRGM1 | Cell Signaling Technology | 14979 |
| anti-DDB1 | BBI Life Sciences | D110058 |
| anti-ROC1 | Abcam | ab133565 |
| anti-Wdr43 | BETHYL | A302-478A-T |
| anti-WDR1 | Abcam | ab173574 |
| anti-WDR77 | Abcam | ab154190 |
| anti-Actin | Santa | SC69879 |
| anti-HA | Rochland | 600-401-384 |
| anti-Ub | Cell Signaling Technology | 3933 |
| anti-pAMPKα(T172) | Cell Signaling Technology | 40H9 |
| anti-Phospho-ULK1 (Ser555) (D1H4) | Cell Signaling Technology | 5869 |
| anti-Phospho-AMPKα (Thr172) (40H9) | Cell Signaling Technology | 2535 |
| anti-Beclin1 | Cell Signaling Technology | 3495 |
| anti-Atg12 | Cell Signaling Technology | 4180 |
| anti-Atg5 | Cell Signaling Technology | 12994 |
| anti-Atg3 | Cell Signaling Technology | 3415 |
| anti-Atg7 | Cell Signaling Technology | 8558 |
| anti-Atg16L1 | Cell Signaling Technology | 8089 |
| anti-LC3A/B | Cell Signaling Technology | 12741 |
| anti-lysozyme | Abcam | ab108508 |
| UEA-1 | Sigma | L9006 |
| anti-CD45 | Abcam | ab10558 |
| Goat polyclonal anti-mouse IgG (H+L), Alexa Fluor 488 | Jackson ImmunoResearch | 115-545-166 |
| Goat polyclonal anti-rabbit IgG (H+L), Alexa Fluor488 | Jackson ImmunoResearch | 111-547-003 |
| Rhodamine (TRITC)–conjugated Goat Anti-Rat IgG(H+L) | Proteintech | SA00007-7 |
| Donkey polyclonal Secondary Antibody to Rabbit IgG - H&L (Alexa Fluor® 594) | Abcam | ab150076 |
| Donkey Anti-Mouse IgG H&L (Alexa Fluor® 488) | Abcam | ab150105 |
| Donkey anti-Goat IgG (H+L) Cross-Adsorbed Secondary Antibody, Alexa Fluor 488 | life technologies | A11055 |
| Donkey anti-Rabbit IgG (H+L) Highly Cross-Adsorbed Secondary Antibody, Alexa Fluor 488 | life technologies | A21206 |
| **Chemicals, Peptides, and Recombinant Proteins** | | |
| Fetal Bovine Serum, Qualified, New Zealand Origin | Gibico | 10091148 |
| Foetal Bovine Serum Excellent | AusGeneX | FBSSA500-S |
| Trypsin-EDTA (0.25%), phenol red | Gibico | 25200072 |
| Advanced DMEM/F12 | Thermo Fisher Scientific | 12634010 |
| HEPES | Thermo Fisher Scientific | 15630080 |
| Penicillin-Streptomycin | Thermo Fisher Scientific | 15140122 |
| GlutaMax | Invitrogen | 35050-068 |
| Matrigel | Corning | 356231 |
| B-27 | Gibco | 1704-044 |
| N-Acetyl-L-cysteine | Sigma-Aldrich | A9165 |
| Recombinant mouse EGF | Peprotech | 100-47 |
| Primocin™ | InvivoGen | ant-pm-1 |
| A83-01 | TOCRIC | 2939 |
| Y-27632 | Selleck | S1049 |
| Nicotinamide | Sigma-Aldrich | N0636 |
| CHIR99021 | SIGMA-ALDRICH | SML1046 |
| IWP-2 | Tocris | 3533 |
| Cell Recovery Solution | Corning | 354253 |
| DPBS | Gibco | C14190500CP |
| Fluoroshield™ with DAPI | Sigma-Aldrich | F6057 |
| Puromycin | Thermo Fisher Scientific | A1113803 |
| Dextran sulfate sodium salt | Sigma-Aldrich | 42867 |
| **Critical Commercial Assays** | | |
| RNeasy Mini Kit | Qiagen | 74014 |
| Dual-Luciferase Reporter Assay System | Promega | E1910 |
| Trizol REAGENT | Invitrogen | 15596018 |
| CellTiter-Glo 3D Cell Viability Assay | Promega | G9683 |
| SuperSignal™ West Femto Trial Kit | Thermo Fisher Scientific | 34095 |
| Trizol reagent | Thermo Fisher Scientific | 15596026 |
